# HR-TEM and FT-Raman dataset of the caffeine interacted Phe–Phe peptide nanotube for possible sensing applications

**DOI:** 10.1016/j.dib.2017.12.003

**Published:** 2017-12-14

**Authors:** A. Lakshmi Narayanan, M. Dhamodaran, J. Samu Solomon, B. Karthikeyan, R. Govindhan

**Affiliations:** a Research & Development Center, Bharathiar University, Coimbatore, Tamil Nadu, India; b Department of Chemistry, Perunthalaivar Kamarajar Institute of Engineering and Technology, Karaikal, Pudhucherry - UT, India; c Department of Chemistry, TBML College, Porayar, Tamil Nadu, India; d Department of Chemistry, Annamalai University, Annamalainagar 608002, Tamil Nadu, India

**Keywords:** Caffeine, PNTs, Sensing, HR-TEM, FT-Raman data

## Abstract

Sensing ability of caffeine interaction with Phe-Phe annotates (PNTs), is presented (Govindhan et al., 2017; Karthikeyan et al., 2014; Tavagnacco et al., 2013; Kennedy et al., 2011; Wang et al., 2017) [1–5] in this data set. Investigation of synthesized caffeine carrying peptide nanotubes are carried out by FT-Raman spectral analysis and high resolution transmission electron microscopy (HR-TEM). Particle size of the caffeine loaded PNTs is < 40 nm. The FT-Raman spectrum signals are enhanced in the region of 400–1700 cm^−1^. These data are ideal tool for the applications like biosensing and drug delivery research (DDS).

## Graphical abstract

**Figure:**
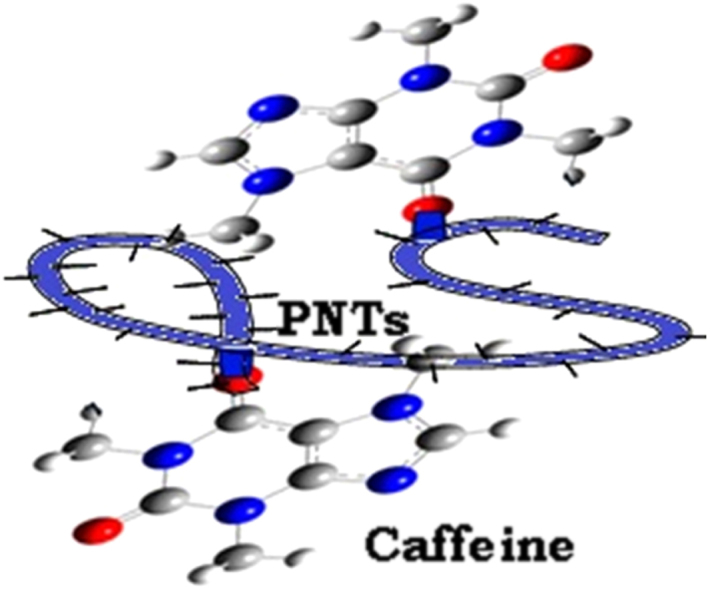

**Specifications Table**

**Table:** 

Subject area	Chemistry
More specific subject area	Biochemistry and Biomaterials
Type of data	Tables, figures and schemes.
How data was acquired	Spectroscopic and microscopic data used to the sensing and drug delivery applications is explored.
Data format	Raw, Partially analyzed.
Experimental factors	FT-Raman and SERS spectra were recorded in an integral microscope Bruker RFS 27 spectrometer equipped with 1024 × 256 pixels liquid nitrogen-cooled germanium detector. A monochromatic beam of incident radiation of wavelength 1064 nm emitted from neodymium doped yttrium aluminium garnet (Nd-YAG) laser was used as an excitation source.
Experimental features	FT-Raman spectral result reveals that sensing potential of PNTs. FT-Raman spectral vibrational features of peptide and caffeine. HR-TEM morphology of as-synthesized caffeine loaded PNTs.
Data source location	Department of Chemistry, Perunthalaivar Kamarajar Institute of Engineering and Technology, Karaikal, Pudhucherry−UT, India.
Data accessibility	The data is available with this article

**Value of the data**

• The data reported here represent a valuable collection of the entire individual spectroscopic and microscopic data of caffeine loaded PNTs [1].
• The microscopic images and plots provide a novel way to look at the effectiveness of the sensing potential of PNTs and further evolutions for other researchers to develop the future outcomes [2].
• The FT-Raman data provides specifying binding frequencies of the caffeine with PNTs [3], [4], [5], [6], [7].
• This data allows other researchers to explore or extend the biosensing and DDS analysis.

## 1 Data

**Fig. 1:**
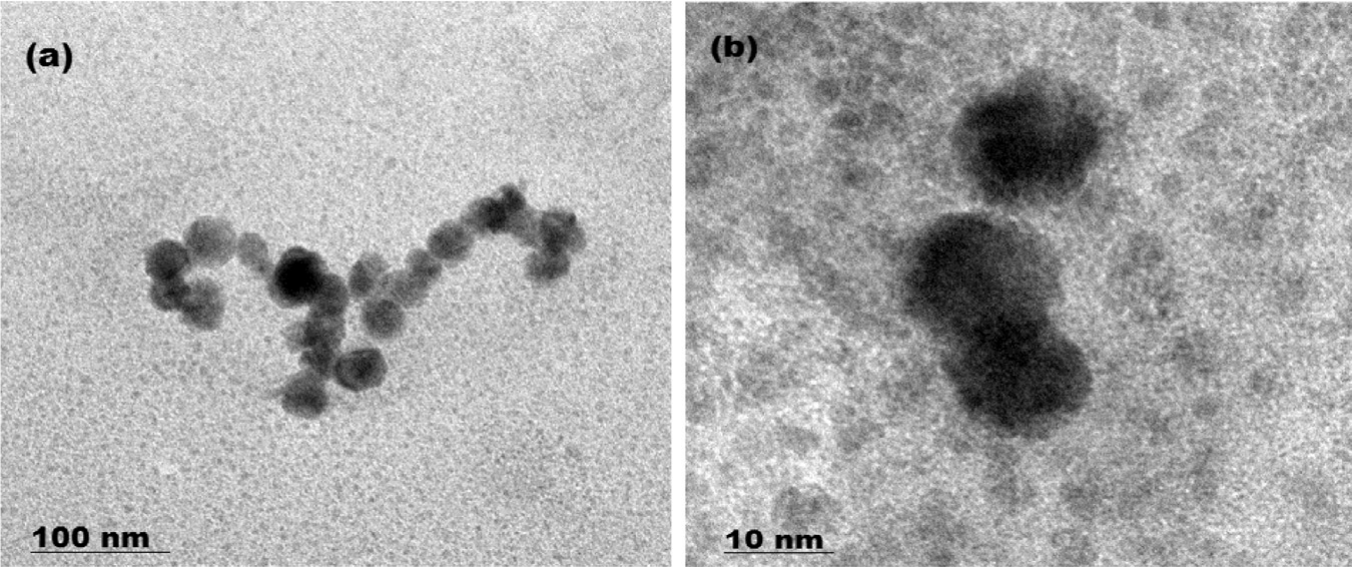
HR-TEM morphologies of BTTPNTs@caffeine (a) 100 nm and (b) 10 nm scale bar.

**Fig. 2:**
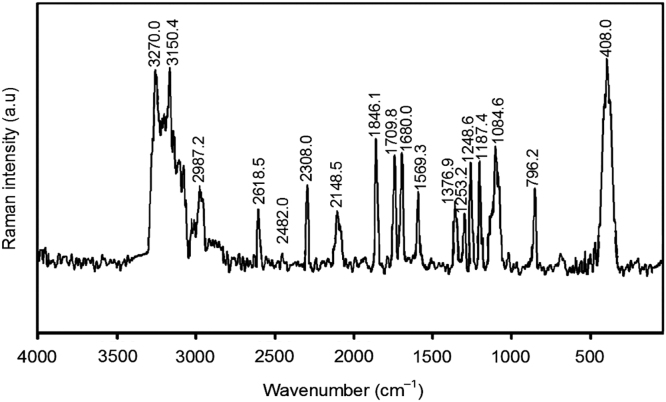
FT-Raman spectrum of BTTPNTs@caffeine.

**Scheme 1:**
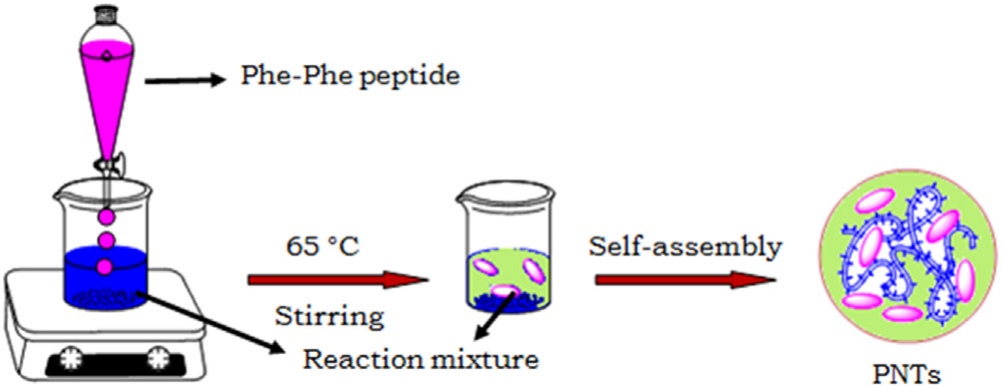
Schematic representation for the synthesis of PNTs and nanovesicles.

**Scheme 2:**
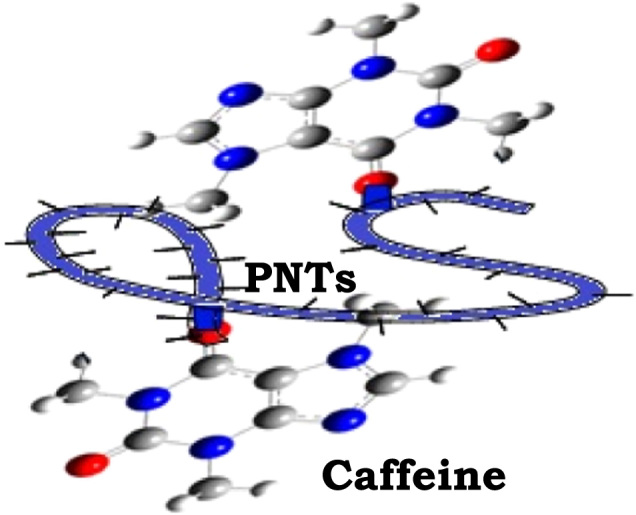
Synthesis of caffeine entrapped PNTs and nanovesicles (PNTs@caffeine).

The data reported [1], [6] includes information about the more information on (Table 1) the HR-TEM data of caffeine interaction with PNTs and Fig. 1 shows the morphology of the reported materials. The Fig. 2, and Scheme 1, Scheme 2 show the synthesis scheme and sensing of caffeine, spectroscopic data in (Table S1). The resulted data are provided in Supplementary Table S1.

**Table 1:** h-TEM data of particle size distribution of caffeine@PNTs.

**S. no.**	**Area (nm)**	**Mean (nm)**	**Min (nm)**	**Max (nm)**	**Angle (deg)**	**Length (nm)**
1	30	118.32	68	228.09	22.17	29.15
2	37	121.74	41.56	212.19	27.98	36.24
3	31	103.11	42.07	198.67	41.01	30.48
4	30	104.2	33.05	212.46	30.96	29.15
5	30	88.35	41	189.67	30.96	29.15
6	33	110.23	41.3	188.68	20.14	31.95
7	29	112.45	52.8	211.96	32.01	28.3
8	31	115.32	59.24	186.28	29.98	30.02
9	31	91.09	46.33	178.67	27.41	30.41
10	32	81.14	41.68	193.67	35.75	30.81
11	33	117.6	65.11	189.51	48.81	31.89
12	32	99.04	51.09	187.67	45	31.11
13	40	61.09	1.29	212.78	37.75	39.2
14	31	111.07	68.47	189.33	25.71	29.97
15	32	156.54	109.04	236.97	33.18	31.06
16	32	63.46	18.01	196.33	35.75	30.81
17	37	68.66	11.5	192.67	41.63	36.12
18	32	110.42	63.49	176.33	58.39	30.53
19	31	93.13	53.17	194	43.67	30.41
20	32	65.01	0.89	197.33	56.82	31.06
21	32	59.62	0.71	166.7	45	31.11
22	32	99.99	44.63	188.67	29.05	30.89

## 2 Experimental design, materials and methods

### 2.1 Materials

Phe–Phe dipeptide, 98.6%, TFA, 98.0%, Caffeine, 98.9%, and other reagents and solvents were purchased from HiMedia Laboratories Pvt. Ltd., (Mumbai, India) it is used as without further purification. All aqueous solutions were prepared with nanopure water. All apparatus and glassware's are washed with acetone, rinsed with deionized water (DIW) and dried with air hot Owen at 100 °C, then it was used throughout the studies.

### 2.2 Synthesis of self-assembled nanotubes and vesicles (PNTs)

The procedure was adopted from a reported method [1] as follows Scheme 1. About 10 mg of aqueous solution (in nanopure water) of single amino acid derivatives was prepared in a closed Erlenmeyer flask with appropriate concentration. The flask containing desired solution and a magnetic stir bar was placed in pre-warmed silicon oil bath (65 °C) and moderate stirring was continued for 30 min at that temperature. Heating was then stopped and the solution was brought to room temperature with gentle stirring for over a period of 3 h.

### 2.3 Synthesis of PNTs entrapment of caffeine (PNTs@caffeine)

A synthetic procedure for loading the peptide nanotubes was briefly outlined in Scheme 2. Briefly, caffeine solution (5 mL) taken in a beaker and stirred for 30 min at room temperature. As-synthesized PNTs sol (1 mL) was added slowly into the beaker containing caffeine solution with drop wise manner. The mixture was stirred for another 30 min at room temperature to form caffeine entrapped PNTs sol, which resulted in the formation of thick sol-gel turbidity containing the PNTs@caffeine.

### 2.4 Characterization techniques

High resolution transmission electron microscopy (HR-TEM) images were recorded using a JEOL 3010 high resolution transmission electron microscopy with an ultra-high resolution (UHR) pole-piece operates at an accelerated voltage of 300 kV. The analyte for HR-TEM studies were prepared by depositing a drop of the synthesized sample on a carbon coated Cu grid and allowing it to evaporate. HR-TEM image was enhanced by using image J viewer software. FT-Raman and SERS spectra were recorded in an integral microscope Bruker RFS 27 spectrometer equipped with 1024 × 256 pixels liquid nitrogen-cooled germanium detector. A monochromatic beam of incident radiation of wavelength 1064 nm emitted from neodymium doped yttrium aluminium garnet (Nd-YAG) laser was used as an excitation source.

Interpretation of FT-Raman spectral data of PNTs@caffeine is mainly focused on the caffeine interaction with PNTs vibrations at 408.0 cm^−^^1^ represent to C-N-O (caffeine) bending, and 3150.4, 3270.0 cm^−^^1^ depict the NH and NH_2_ stretching vibrations is confirmed by the caffeine adsorbed on the PNTs surfaces. The SERS regions at 400–1800 cm^-1^ are CH, CH_2_ bending vibrations and CO stretching vibrations respectively [2], [5], [7] (see Supporting information Table S1).
