# Supplementary material for: HR-TEM and FT-Raman dataset of the caffeine interacted Phe–Phe peptide nanotube for possible sensing applications
Source: Data Brief. 2017 Dec 14;16:1051–5. doi: 10.1016/j.dib.2017.12.003 (PMC5758923; doi:10.1016/j.dib.2017.12.003)
Supplement: Supplementary file 1 — Transparency document [file mmc1.docx]

**Conflict of Interest**

The authors declare no competing financial interest.
