# Supplementary material for: HR-TEM and FT-Raman dataset of the caffeine interacted Phe–Phe peptide nanotube for possible sensing applications
Source: Data Brief. 2017 Dec 14;16:1051–5. doi: 10.1016/j.dib.2017.12.003 (PMC5758923; doi:10.1016/j.dib.2017.12.003)
Supplement: Supplementary file 2 — Supplementary material [file mmc2.doc]

**Supplementary Material**

**HR-TEM and FT-Raman dataset of the caffeine interacted Phe−Phe peptide nanotube for possible sensing applications**

1. Lakshmi Narayanan,^abcd^* M. Dhamodaran,^ab^ J. Samu Solomon,^bc^ B. Karthikeyan^d^ and
   R. Govindhan^d^

^a^  Research & Development Center, Bharathiar University, Coimbatore, Tamilnadu, India.

^b^ Department of Chemistry, Perunthalaivar Kamarajar Institute of Engineering and Technology, Karaikal, Pudhucherry−UT, India.

^c^ Department of Chemistry, TBML College, Porayar, Tamilnadu, India.

^d^ Department of Chemistry, Annamalai University, Annamalainagar 608002, Tamilnadu, India.

**Table S1.** FT-Raman spectral data of PNTs@caffeine.

| **Wave number**  **(cm^−1^)** | **Raman intensity (a.u)** |
| --- | --- |
| 3600.313 | 0.00189 |
| 3599.349 | 0.00189 |
| 3598.385 | 0.00189 |
| 3597.421 | 0.00189 |
| 3596.456 | 0.00189 |
| 3595.492 | 0.00189 |
| 3594.528 | 0.00189 |
| 3593.564 | 0.00189 |
| 3592.599 | 0.00189 |
| 3591.635 | 0.00189 |
| 3590.671 | 0.00189 |
| 3589.706 | 0.00189 |
| 3588.742 | 0.00189 |
| 3587.778 | 0.00185 |
| 3586.814 | 0.00181 |
| 3585.849 | 0.00171 |
| 3584.885 | 0.00152 |
| 3583.921 | 0.00135 |
| 3582.957 | 0.00119 |
| 3581.992 | 0.00107 |
| 3581.028 | 0.00099 |
| 3580.064 | 0.00093 |
| 3579.1 | 0.00089 |
| 3578.135 | 0.00088 |
| 3577.171 | 0.00088 |
| 3576.207 | 0.00086 |
| 3575.242 | 0.00086 |
| 3574.278 | 0.00086 |
| 3573.314 | 0.00091 |
| 3572.35 | 0.00101 |
| 3571.385 | 0.00118 |
| 3570.421 | 0.0014 |
| 3569.457 | 0.00155 |
| 3568.493 | 0.00161 |
| 3567.528 | 0.00167 |
| 3566.564 | 0.00174 |
| 3565.6 | 0.00172 |
| 3564.636 | 0.00167 |
| 3563.671 | 0.00162 |
| 3562.707 | 0.00156 |
| 3561.743 | 0.00148 |
| 3560.778 | 0.00144 |
| 3559.814 | 0.00143 |
| 3558.85 | 0.00135 |
| 3557.886 | 0.00127 |
| 3556.921 | 0.00121 |
| 3555.957 | 0.00118 |
| 3554.993 | 0.00118 |
| 3554.029 | 0.00117 |
| 3553.064 | 0.00112 |
| 3552.1 | 0.00111 |
| 3551.136 | 0.00117 |
| 3550.172 | 0.0013 |
| 3549.207 | 0.0014 |
| 3548.243 | 0.00146 |
| 3547.279 | 0.00149 |
| 3546.314 | 0.00149 |
| 3545.35 | 0.00148 |
| 3544.386 | 0.00146 |
| 3543.422 | 0.00141 |
| 3542.457 | 0.00126 |
| 3541.493 | 0.00109 |
| 3540.529 | 0.00099 |
| 3539.565 | 0.00099 |
| 3538.6 | 0.00096 |
| 3537.636 | 0.00093 |
| 3536.672 | 0.00091 |
| 3535.707 | 0.00084 |
| 3534.743 | 0.00083 |
| 3533.779 | 0.00081 |
| 3532.815 | 0.0008 |
| 3531.85 | 0.00081 |
| 3530.886 | 0.00079 |
| 3529.922 | 0.00075 |
| 3528.958 | 0.00077 |
| 3527.993 | 0.00089 |
| 3527.029 | 0.00103 |
| 3526.065 | 0.00115 |
| 3525.101 | 0.00125 |
| 3524.136 | 0.00133 |
| 3523.172 | 0.00141 |
| 3522.208 | 0.00151 |
| 3521.243 | 0.00156 |
| 3520.279 | 0.00158 |
| 3519.315 | 0.00163 |
| 3518.351 | 0.00171 |
| 3517.386 | 0.0018 |
| 3516.422 | 0.00191 |
| 3515.458 | 0.00196 |
| 3514.494 | 0.002 |
| 3513.529 | 0.00201 |
| 3512.565 | 0.00197 |
| 3511.601 | 0.00187 |
| 3510.637 | 0.00174 |
| 3509.672 | 0.0016 |
| 3508.708 | 0.00151 |
| 3507.744 | 0.00145 |
| 3506.779 | 0.00137 |
| 3505.815 | 0.00131 |
| 3504.851 | 0.00123 |
| 3503.887 | 0.00108 |
| 3502.922 | 0.00094 |
| 3501.958 | 0.00087 |
| 3500.994 | 0.00089 |
| 3500.03 | 0.00099 |
| 3499.065 | 0.00111 |
| 3498.101 | 0.00123 |
| 3497.137 | 0.00138 |
| 3496.172 | 0.00155 |
| 3495.208 | 0.00169 |
| 3494.244 | 0.00181 |
| 3493.28 | 0.00188 |
| 3492.315 | 0.0019 |
| 3491.351 | 0.00194 |
| 3490.387 | 0.00201 |
| 3489.423 | 0.00206 |
| 3488.458 | 0.00204 |
| 3487.494 | 0.00194 |
| 3486.53 | 0.00185 |
| 3485.566 | 0.00182 |
| 3484.601 | 0.00178 |
| 3483.637 | 0.00162 |
| 3482.673 | 0.00145 |
| 3481.708 | 0.00132 |
| 3480.744 | 0.00123 |
| 3479.78 | 0.00121 |
| 3478.816 | 0.00126 |
| 3477.851 | 0.00132 |
| 3476.887 | 0.00137 |
| 3475.923 | 0.0014 |
| 3474.959 | 0.00142 |
| 3473.994 | 0.00143 |
| 3473.03 | 0.00144 |
| 3472.066 | 0.00142 |
| 3471.102 | 0.00138 |
| 3470.137 | 0.00135 |
| 3469.173 | 0.00139 |
| 3468.209 | 0.00149 |
| 3467.244 | 0.00159 |
| 3466.28 | 0.00162 |
| 3465.316 | 0.0016 |
| 3464.352 | 0.00159 |
| 3463.387 | 0.00167 |
| 3462.423 | 0.0018 |
| 3461.459 | 0.00192 |
| 3460.495 | 0.00201 |
| 3459.53 | 0.00208 |
| 3458.566 | 0.00214 |
| 3457.602 | 0.00214 |
| 3456.638 | 0.00206 |
| 3455.673 | 0.00198 |
| 3454.709 | 0.00196 |
| 3453.745 | 0.00195 |
| 3452.78 | 0.00188 |
| 3451.816 | 0.0018 |
| 3450.852 | 0.00168 |
| 3449.888 | 0.00155 |
| 3448.923 | 0.00149 |
| 3447.959 | 0.00152 |
| 3446.995 | 0.00154 |
| 3446.031 | 0.00146 |
| 3445.066 | 0.00135 |
| 3444.102 | 0.00128 |
| 3443.138 | 0.00127 |
| 3442.173 | 0.00129 |
| 3441.209 | 0.00135 |
| 3440.245 | 0.00145 |
| 3439.281 | 0.00158 |
| 3438.316 | 0.00172 |
| 3437.352 | 0.0018 |
| 3436.388 | 0.00182 |
| 3435.424 | 0.0018 |
| 3434.459 | 0.00173 |
| 3433.495 | 0.00168 |
| 3432.531 | 0.00176 |
| 3431.567 | 0.00193 |
| 3430.602 | 0.0021 |
| 3429.638 | 0.0022 |
| 3428.674 | 0.00227 |
| 3427.709 | 0.00224 |
| 3426.745 | 0.00221 |
| 3425.781 | 0.0023 |
| 3424.817 | 0.00241 |
| 3423.852 | 0.00236 |
| 3422.888 | 0.00226 |
| 3421.924 | 0.00226 |
| 3420.96 | 0.00231 |
| 3419.995 | 0.00233 |
| 3419.031 | 0.00232 |
| 3418.067 | 0.00233 |
| 3417.103 | 0.00235 |
| 3416.138 | 0.00237 |
| 3415.174 | 0.00237 |
| 3414.21 | 0.00232 |
| 3413.245 | 0.00225 |
| 3412.281 | 0.00221 |
| 3411.317 | 0.00222 |
| 3410.353 | 0.00229 |
| 3409.388 | 0.00242 |
| 3408.424 | 0.00255 |
| 3407.46 | 0.00264 |
| 3406.496 | 0.0027 |
| 3405.531 | 0.00269 |
| 3404.567 | 0.00261 |
| 3403.603 | 0.00254 |
| 3402.639 | 0.0025 |
| 3401.674 | 0.00241 |
| 3400.71 | 0.00226 |
| 3399.746 | 0.0021 |
| 3398.781 | 0.00197 |
| 3397.817 | 0.00183 |
| 3396.853 | 0.0017 |
| 3395.889 | 0.00155 |
| 3394.924 | 0.00142 |
| 3393.96 | 0.00129 |
| 3392.996 | 0.00119 |
| 3392.032 | 0.00114 |
| 3391.067 | 0.00117 |
| 3390.103 | 0.00126 |
| 3389.139 | 0.00137 |
| 3388.174 | 0.00144 |
| 3387.21 | 0.00149 |
| 3386.246 | 0.00155 |
| 3385.282 | 0.00165 |
| 3384.317 | 0.0018 |
| 3383.353 | 0.00198 |
| 3382.389 | 0.00205 |
| 3381.425 | 0.00206 |
| 3380.46 | 0.00204 |
| 3379.496 | 0.00206 |
| 3378.532 | 0.0022 |
| 3377.568 | 0.00241 |
| 3376.603 | 0.00265 |
| 3375.639 | 0.00285 |
| 3374.675 | 0.00296 |
| 3373.71 | 0.00299 |
| 3372.746 | 0.00298 |
| 3371.782 | 0.00298 |
| 3370.818 | 0.00298 |
| 3369.853 | 0.00299 |
| 3368.889 | 0.00301 |
| 3367.925 | 0.00303 |
| 3366.961 | 0.00306 |
| 3365.996 | 0.00307 |
| 3365.032 | 0.00305 |
| 3364.068 | 0.00302 |
| 3363.104 | 0.00305 |
| 3362.139 | 0.00312 |
| 3361.175 | 0.00317 |
| 3360.211 | 0.00315 |
| 3359.246 | 0.00308 |
| 3358.282 | 0.00306 |
| 3357.318 | 0.00309 |
| 3356.354 | 0.00322 |
| 3355.389 | 0.00338 |
| 3354.425 | 0.00349 |
| 3353.461 | 0.00348 |
| 3352.497 | 0.00339 |
| 3351.532 | 0.00327 |
| 3350.568 | 0.00319 |
| 3349.604 | 0.00318 |
| 3348.64 | 0.00322 |
| 3347.675 | 0.00327 |
| 3346.711 | 0.00332 |
| 3345.747 | 0.00333 |
| 3344.782 | 0.00335 |
| 3343.818 | 0.00341 |
| 3342.854 | 0.00351 |
| 3341.89 | 0.00361 |
| 3340.925 | 0.00372 |
| 3339.961 | 0.00381 |
| 3338.997 | 0.00384 |
| 3338.033 | 0.00383 |
| 3337.068 | 0.00389 |
| 3336.104 | 0.00401 |
| 3335.14 | 0.00414 |
| 3334.175 | 0.00424 |
| 3333.211 | 0.00426 |
| 3332.247 | 0.00419 |
| 3331.283 | 0.00404 |
| 3330.318 | 0.00383 |
| 3329.354 | 0.00363 |
| 3328.39 | 0.00352 |
| 3327.426 | 0.00351 |
| 3326.461 | 0.00359 |
| 3325.497 | 0.00368 |
| 3324.533 | 0.00371 |
| 3323.569 | 0.00365 |
| 3322.604 | 0.00354 |
| 3321.64 | 0.00344 |
| 3320.676 | 0.00337 |
| 3319.711 | 0.00332 |
| 3318.747 | 0.00328 |
| 3317.783 | 0.00324 |
| 3316.819 | 0.00323 |
| 3315.854 | 0.00325 |
| 3314.89 | 0.00332 |
| 3313.926 | 0.00338 |
| 3312.962 | 0.00338 |
| 3311.997 | 0.00326 |
| 3311.033 | 0.00303 |
| 3310.069 | 0.00275 |
| 3309.105 | 0.00249 |
| 3308.14 | 0.00227 |
| 3307.176 | 0.00214 |
| 3306.212 | 0.00214 |
| 3305.247 | 0.0023 |
| 3304.283 | 0.00253 |
| 3303.319 | 0.00275 |
| 3302.355 | 0.00292 |
| 3301.39 | 0.00306 |
| 3300.426 | 0.00317 |
| 3299.462 | 0.00327 |
| 3298.498 | 0.00336 |
| 3297.533 | 0.00345 |
| 3296.569 | 0.00357 |
| 3295.605 | 0.00371 |
| 3294.64 | 0.00387 |
| 3293.676 | 0.00398 |
| 3292.712 | 0.00398 |
| 3291.748 | 0.00388 |
| 3290.783 | 0.00374 |
| 3289.819 | 0.00359 |
| 3288.855 | 0.00343 |
| 3287.891 | 0.00332 |
| 3286.926 | 0.00334 |
| 3285.962 | 0.0035 |
| 3284.998 | 0.00368 |
| 3284.034 | 0.00377 |
| 3283.069 | 0.00384 |
| 3282.105 | 0.00391 |
| 3281.141 | 0.00397 |
| 3280.176 | 0.00401 |
| 3279.212 | 0.00402 |
| 3278.248 | 0.00402 |
| 3277.284 | 0.00406 |
| 3276.319 | 0.00414 |
| 3275.355 | 0.00427 |
| 3274.391 | 0.00444 |
| 3273.427 | 0.00461 |
| 3272.462 | 0.00471 |
| 3271.498 | 0.00473 |
| 3270.534 | 0.00467 |
| 3269.57 | 0.00458 |
| 3268.605 | 0.00457 |
| 3267.641 | 0.00467 |
| 3266.677 | 0.00484 |
| 3265.712 | 0.00502 |
| 3264.748 | 0.00525 |
| 3263.784 | 0.00552 |
| 3262.82 | 0.00578 |
| 3261.855 | 0.00596 |
| 3260.891 | 0.00605 |
| 3259.927 | 0.00609 |
| 3258.963 | 0.00608 |
| 3257.998 | 0.00603 |
| 3257.034 | 0.00594 |
| 3256.07 | 0.00583 |
| 3255.106 | 0.00574 |
| 3254.141 | 0.00572 |
| 3253.177 | 0.00579 |
| 3252.213 | 0.0059 |
| 3251.248 | 0.00597 |
| 3250.284 | 0.00592 |
| 3249.32 | 0.00575 |
| 3248.356 | 0.00552 |
| 3247.391 | 0.00529 |
| 3246.427 | 0.00516 |
| 3245.463 | 0.00518 |
| 3244.499 | 0.00528 |
| 3243.534 | 0.00536 |
| 3242.57 | 0.00536 |
| 3241.606 | 0.00535 |
| 3240.641 | 0.00537 |
| 3239.677 | 0.00545 |
| 3238.713 | 0.00556 |
| 3237.749 | 0.0056 |
| 3236.784 | 0.00557 |
| 3235.82 | 0.00549 |
| 3234.856 | 0.00547 |
| 3233.892 | 0.00551 |
| 3232.927 | 0.00552 |
| 3231.963 | 0.00549 |
| 3230.999 | 0.00545 |
| 3230.035 | 0.00544 |
| 3229.07 | 0.0054 |
| 3228.106 | 0.00531 |
| 3227.142 | 0.0052 |
| 3226.177 | 0.00506 |
| 3225.213 | 0.00488 |
| 3224.249 | 0.0047 |
| 3223.285 | 0.0046 |
| 3222.32 | 0.00459 |
| 3221.356 | 0.00458 |
| 3220.392 | 0.00453 |
| 3219.428 | 0.00449 |
| 3218.463 | 0.00448 |
| 3217.499 | 0.00451 |
| 3216.535 | 0.00458 |
| 3215.571 | 0.00469 |
| 3214.606 | 0.00479 |
| 3213.642 | 0.00488 |
| 3212.678 | 0.00499 |
| 3211.713 | 0.00518 |
| 3210.749 | 0.00543 |
| 3209.785 | 0.00563 |
| 3208.821 | 0.00574 |
| 3207.856 | 0.0058 |
| 3206.892 | 0.00586 |
| 3205.928 | 0.00594 |
| 3204.964 | 0.00602 |
| 3203.999 | 0.00605 |
| 3203.035 | 0.00599 |
| 3202.071 | 0.00588 |
| 3201.107 | 0.0058 |
| 3200.142 | 0.0058 |
| 3199.178 | 0.00584 |
| 3198.214 | 0.00585 |
| 3197.249 | 0.00582 |
| 3196.285 | 0.00575 |
| 3195.321 | 0.00568 |
| 3194.357 | 0.0056 |
| 3193.392 | 0.0055 |
| 3192.428 | 0.00536 |
| 3191.464 | 0.00518 |
| 3190.5 | 0.005 |
| 3189.535 | 0.00488 |
| 3188.571 | 0.00486 |
| 3187.607 | 0.00492 |
| 3186.642 | 0.00497 |
| 3185.678 | 0.0049 |
| 3184.714 | 0.00479 |
| 3183.75 | 0.00475 |
| 3182.785 | 0.0048 |
| 3181.821 | 0.00484 |
| 3180.857 | 0.00481 |
| 3179.893 | 0.00474 |
| 3178.928 | 0.00469 |
| 3177.964 | 0.00475 |
| 3177 | 0.00491 |
| 3176.036 | 0.00511 |
| 3175.071 | 0.0053 |
| 3174.107 | 0.00549 |
| 3173.143 | 0.00571 |
| 3172.178 | 0.00589 |
| 3171.214 | 0.00599 |
| 3170.25 | 0.00603 |
| 3169.286 | 0.00604 |
| 3168.321 | 0.00609 |
| 3167.357 | 0.00614 |
| 3166.393 | 0.00613 |
| 3165.429 | 0.00605 |
| 3164.464 | 0.00588 |
| 3163.5 | 0.00565 |
| 3162.536 | 0.00543 |
| 3161.572 | 0.0053 |
| 3160.607 | 0.00523 |
| 3159.643 | 0.00511 |
| 3158.679 | 0.00489 |
| 3157.714 | 0.00461 |
| 3156.75 | 0.00435 |
| 3155.786 | 0.00419 |
| 3154.822 | 0.00416 |
| 3153.857 | 0.00418 |
| 3152.893 | 0.00417 |
| 3151.929 | 0.0041 |
| 3150.965 | 0.00403 |
| 3150 | 0.00402 |
| 3149.036 | 0.00408 |
| 3148.072 | 0.00419 |
| 3147.107 | 0.00431 |
| 3146.143 | 0.00442 |
| 3145.179 | 0.00453 |
| 3144.215 | 0.00458 |
| 3143.25 | 0.00454 |
| 3142.286 | 0.00441 |
| 3141.322 | 0.00428 |
| 3140.358 | 0.00423 |
| 3139.393 | 0.0043 |
| 3138.429 | 0.0044 |
| 3137.465 | 0.00441 |
| 3136.501 | 0.00432 |
| 3135.536 | 0.00419 |
| 3134.572 | 0.00401 |
| 3133.608 | 0.00385 |
| 3132.643 | 0.00373 |
| 3131.679 | 0.00365 |
| 3130.715 | 0.00361 |
| 3129.751 | 0.00355 |
| 3128.786 | 0.00349 |
| 3127.822 | 0.00348 |
| 3126.858 | 0.00356 |
| 3125.894 | 0.00365 |
| 3124.929 | 0.00369 |
| 3123.965 | 0.00361 |
| 3123.001 | 0.00352 |
| 3122.037 | 0.00351 |
| 3121.072 | 0.00357 |
| 3120.108 | 0.00363 |
| 3119.144 | 0.00367 |
| 3118.179 | 0.00373 |
| 3117.215 | 0.00383 |
| 3116.251 | 0.0039 |
| 3115.287 | 0.00395 |
| 3114.322 | 0.00399 |
| 3113.358 | 0.00409 |
| 3112.394 | 0.00423 |
| 3111.43 | 0.00435 |
| 3110.465 | 0.00445 |
| 3109.501 | 0.00455 |
| 3108.537 | 0.00462 |
| 3107.573 | 0.00464 |
| 3106.608 | 0.00461 |
| 3105.644 | 0.00457 |
| 3104.68 | 0.00453 |
| 3103.715 | 0.00446 |
| 3102.751 | 0.0043 |
| 3101.787 | 0.00405 |
| 3100.823 | 0.00379 |
| 3099.858 | 0.00363 |
| 3098.894 | 0.00355 |
| 3097.93 | 0.00347 |
| 3096.966 | 0.00335 |
| 3096.001 | 0.00324 |
| 3095.037 | 0.00317 |
| 3094.073 | 0.00309 |
| 3093.108 | 0.00302 |
| 3092.144 | 0.00302 |
| 3091.18 | 0.00314 |
| 3090.216 | 0.0033 |
| 3089.251 | 0.00341 |
| 3088.287 | 0.00346 |
| 3087.323 | 0.00348 |
| 3086.359 | 0.00348 |
| 3085.394 | 0.00347 |
| 3084.43 | 0.00348 |
| 3083.466 | 0.00351 |
| 3082.502 | 0.00358 |
| 3081.537 | 0.00364 |
| 3080.573 | 0.00369 |
| 3079.609 | 0.00369 |
| 3078.644 | 0.00364 |
| 3077.68 | 0.00361 |
| 3076.716 | 0.00356 |
| 3075.752 | 0.00343 |
| 3074.787 | 0.00326 |
| 3073.823 | 0.00312 |
| 3072.859 | 0.00298 |
| 3071.895 | 0.00283 |
| 3070.93 | 0.0027 |
| 3069.966 | 0.00265 |
| 3069.002 | 0.00268 |
| 3068.038 | 0.00275 |
| 3067.073 | 0.00281 |
| 3066.109 | 0.00284 |
| 3065.145 | 0.00283 |
| 3064.18 | 0.00276 |
| 3063.216 | 0.00264 |
| 3062.252 | 0.0025 |
| 3061.288 | 0.00236 |
| 3060.323 | 0.00225 |
| 3059.359 | 0.00219 |
| 3058.395 | 0.0021 |
| 3057.431 | 0.00197 |
| 3056.466 | 0.00184 |
| 3055.502 | 0.00177 |
| 3054.538 | 0.00175 |
| 3053.574 | 0.00168 |
| 3052.609 | 0.00159 |
| 3051.645 | 0.00156 |
| 3050.681 | 0.00148 |
| 3049.716 | 0.00139 |
| 3048.752 | 0.00137 |
| 3047.788 | 0.00149 |
| 3046.824 | 0.00167 |
| 3045.859 | 0.00178 |
| 3044.895 | 0.00182 |
| 3043.931 | 0.00182 |
| 3042.967 | 0.00178 |
| 3042.002 | 0.00171 |
| 3041.038 | 0.00163 |
| 3040.074 | 0.00153 |
| 3039.109 | 0.00145 |
| 3038.145 | 0.00143 |
| 3037.181 | 0.00147 |
| 3036.217 | 0.00152 |
| 3035.252 | 0.00155 |
| 3034.288 | 0.00161 |
| 3033.324 | 0.00174 |
| 3032.36 | 0.00188 |
| 3031.395 | 0.002 |
| 3030.431 | 0.00205 |
| 3029.467 | 0.00209 |
| 3028.503 | 0.00208 |
| 3027.538 | 0.00198 |
| 3026.574 | 0.00195 |
| 3025.61 | 0.00204 |
| 3024.645 | 0.00218 |
| 3023.681 | 0.00223 |
| 3022.717 | 0.00216 |
| 3021.753 | 0.00208 |
| 3020.788 | 0.00205 |
| 3019.824 | 0.00202 |
| 3018.86 | 0.00195 |
| 3017.896 | 0.00185 |
| 3016.931 | 0.00179 |
| 3015.967 | 0.00184 |
| 3015.003 | 0.00196 |
| 3014.039 | 0.00207 |
| 3013.074 | 0.00214 |
| 3012.11 | 0.00218 |
| 3011.146 | 0.00217 |
| 3010.181 | 0.00215 |
| 3009.217 | 0.00216 |
| 3008.253 | 0.0022 |
| 3007.289 | 0.00223 |
| 3006.324 | 0.00225 |
| 3005.36 | 0.0023 |
| 3004.396 | 0.00244 |
| 3003.432 | 0.00261 |
| 3002.467 | 0.00276 |
| 3001.503 | 0.00283 |
| 3000.539 | 0.00286 |
| 2999.575 | 0.00287 |
| 2998.61 | 0.00288 |
| 2997.646 | 0.00291 |
| 2996.682 | 0.00288 |
| 2995.717 | 0.00279 |
| 2994.753 | 0.00274 |
| 2993.789 | 0.00276 |
| 2992.825 | 0.00276 |
| 2991.86 | 0.00268 |
| 2990.896 | 0.00255 |
| 2989.932 | 0.00242 |
| 2988.968 | 0.0023 |
| 2988.003 | 0.0022 |
| 2987.039 | 0.00214 |
| 2986.075 | 0.00209 |
| 2985.11 | 0.00202 |
| 2984.146 | 0.00195 |
| 2983.182 | 0.00195 |
| 2982.218 | 0.00201 |
| 2981.253 | 0.00209 |
| 2980.289 | 0.00213 |
| 2979.325 | 0.00215 |
| 2978.361 | 0.00216 |
| 2977.396 | 0.00219 |
| 2976.432 | 0.00223 |
| 2975.468 | 0.00227 |
| 2974.504 | 0.0023 |
| 2973.539 | 0.00228 |
| 2972.575 | 0.00223 |
| 2971.611 | 0.00218 |
| 2970.646 | 0.00212 |
| 2969.682 | 0.00206 |
| 2968.718 | 0.00204 |
| 2967.754 | 0.00203 |
| 2966.789 | 0.00199 |
| 2965.825 | 0.00192 |
| 2964.861 | 0.00187 |
| 2963.897 | 0.00186 |
| 2962.932 | 0.00187 |
| 2961.968 | 0.00192 |
| 2961.004 | 0.00201 |
| 2960.04 | 0.0021 |
| 2959.075 | 0.00218 |
| 2958.111 | 0.00224 |
| 2957.147 | 0.00223 |
| 2956.182 | 0.00219 |
| 2955.218 | 0.00218 |
| 2954.254 | 0.00221 |
| 2953.29 | 0.00229 |
| 2952.325 | 0.00239 |
| 2951.361 | 0.00247 |
| 2950.397 | 0.00249 |
| 2949.433 | 0.00248 |
| 2948.468 | 0.00249 |
| 2947.504 | 0.00256 |
| 2946.54 | 0.00266 |
| 2945.575 | 0.00275 |
| 2944.611 | 0.00275 |
| 2943.647 | 0.00274 |
| 2942.683 | 0.00278 |
| 2941.718 | 0.00289 |
| 2940.754 | 0.003 |
| 2939.79 | 0.00308 |
| 2938.826 | 0.00314 |
| 2937.861 | 0.00313 |
| 2936.897 | 0.00306 |
| 2935.933 | 0.00297 |
| 2934.969 | 0.00289 |
| 2934.004 | 0.00285 |
| 2933.04 | 0.00287 |
| 2932.076 | 0.00293 |
| 2931.111 | 0.00298 |
| 2930.147 | 0.003 |
| 2929.183 | 0.00296 |
| 2928.219 | 0.00289 |
| 2927.254 | 0.00282 |
| 2926.29 | 0.00276 |
| 2925.326 | 0.00277 |
| 2924.362 | 0.00282 |
| 2923.397 | 0.00285 |
| 2922.433 | 0.00281 |
| 2921.469 | 0.00276 |
| 2920.505 | 0.00275 |
| 2919.54 | 0.00277 |
| 2918.576 | 0.00281 |
| 2917.612 | 0.00282 |
| 2916.647 | 0.00279 |
| 2915.683 | 0.00273 |
| 2914.719 | 0.00266 |
| 2913.755 | 0.00262 |
| 2912.79 | 0.00259 |
| 2911.826 | 0.00254 |
| 2910.862 | 0.00246 |
| 2909.898 | 0.00232 |
| 2908.933 | 0.00219 |
| 2907.969 | 0.0021 |
| 2907.005 | 0.00207 |
| 2906.041 | 0.00209 |
| 2905.076 | 0.00212 |
| 2904.112 | 0.00216 |
| 2903.148 | 0.00223 |
| 2902.183 | 0.00238 |
| 2901.219 | 0.00254 |
| 2900.255 | 0.00264 |
| 2899.291 | 0.00268 |
| 2898.326 | 0.00268 |
| 2897.362 | 0.00267 |
| 2896.398 | 0.00267 |
| 2895.434 | 0.00271 |
| 2894.469 | 0.00274 |
| 2893.505 | 0.00275 |
| 2892.541 | 0.00274 |
| 2891.576 | 0.0027 |
| 2890.612 | 0.00259 |
| 2889.648 | 0.00244 |
| 2888.684 | 0.00231 |
| 2887.719 | 0.00225 |
| 2886.755 | 0.00225 |
| 2885.791 | 0.00231 |
| 2884.827 | 0.00243 |
| 2883.862 | 0.00254 |
| 2882.898 | 0.00263 |
| 2881.934 | 0.00272 |
| 2880.97 | 0.0028 |
| 2880.005 | 0.00289 |
| 2879.041 | 0.00293 |
| 2878.077 | 0.00287 |
| 2877.112 | 0.00273 |
| 2876.148 | 0.00259 |
| 2875.184 | 0.00245 |
| 2874.22 | 0.00229 |
| 2873.255 | 0.00213 |
| 2872.291 | 0.002 |
| 2871.327 | 0.00184 |
| 2870.363 | 0.00164 |
| 2869.398 | 0.00148 |
| 2868.434 | 0.00137 |
| 2867.47 | 0.00132 |
| 2866.506 | 0.00134 |
| 2865.541 | 0.00142 |
| 2864.577 | 0.00149 |
| 2863.613 | 0.00152 |
| 2862.648 | 0.00158 |
| 2861.684 | 0.00169 |
| 2860.72 | 0.00183 |
| 2859.756 | 0.00188 |
| 2858.791 | 0.00186 |
| 2857.827 | 0.00181 |
| 2856.863 | 0.00178 |
| 2855.899 | 0.00175 |
| 2854.934 | 0.00169 |
| 2853.97 | 0.00162 |
| 2853.006 | 0.00159 |
| 2852.042 | 0.00153 |
| 2851.077 | 0.00144 |
| 2850.113 | 0.0014 |
| 2849.149 | 0.00137 |
| 2848.184 | 0.0013 |
| 2847.22 | 0.00126 |
| 2846.256 | 0.0013 |
| 2845.292 | 0.00138 |
| 2844.327 | 0.0015 |
| 2843.363 | 0.00162 |
| 2842.399 | 0.00173 |
| 2841.435 | 0.00187 |
| 2840.47 | 0.00201 |
| 2839.506 | 0.00218 |
| 2838.542 | 0.00228 |
| 2837.577 | 0.0023 |
| 2836.613 | 0.00225 |
| 2835.649 | 0.00218 |
| 2834.685 | 0.00209 |
| 2833.72 | 0.00199 |
| 2832.756 | 0.00188 |
| 2831.792 | 0.00177 |
| 2830.828 | 0.00168 |
| 2829.863 | 0.0016 |
| 2828.899 | 0.00147 |
| 2827.935 | 0.00131 |
| 2826.971 | 0.00114 |
| 2826.006 | 0.00098 |
| 2825.042 | 0.00085 |
| 2824.078 | 0.00074 |
| 2823.113 | 0.0007 |
| 2822.149 | 0.00073 |
| 2821.185 | 0.00079 |
| 2820.221 | 0.00086 |
| 2819.256 | 0.00096 |
| 2818.292 | 0.00111 |
| 2817.328 | 0.00122 |
| 2816.364 | 0.00131 |
| 2815.399 | 0.00134 |
| 2814.435 | 0.00137 |
| 2813.471 | 0.00142 |
| 2812.507 | 0.00147 |
| 2811.542 | 0.00155 |
| 2810.578 | 0.00166 |
| 2809.614 | 0.00177 |
| 2808.649 | 0.00189 |
| 2807.685 | 0.0019 |
| 2806.721 | 0.00185 |
| 2805.757 | 0.00183 |
| 2804.792 | 0.00186 |
| 2803.828 | 0.00183 |
| 2802.864 | 0.00179 |
| 2801.9 | 0.00176 |
| 2800.935 | 0.00171 |
| 2799.971 | 0.00171 |
| 2799.007 | 0.00174 |
| 2798.042 | 0.00176 |
| 2797.078 | 0.0018 |
| 2796.114 | 0.00185 |
| 2795.15 | 0.00189 |
| 2794.185 | 0.0019 |
| 2793.221 | 0.00188 |
| 2792.257 | 0.00186 |
| 2791.293 | 0.00185 |
| 2790.328 | 0.00185 |
| 2789.364 | 0.00184 |
| 2788.4 | 0.00181 |
| 2787.436 | 0.00175 |
| 2786.471 | 0.0017 |
| 2785.507 | 0.00166 |
| 2784.543 | 0.00158 |
| 2783.578 | 0.00157 |
| 2782.614 | 0.00157 |
| 2781.65 | 0.00148 |
| 2780.686 | 0.00134 |
| 2779.721 | 0.00128 |
| 2778.757 | 0.00129 |
| 2777.793 | 0.00132 |
| 2776.829 | 0.00136 |
| 2775.864 | 0.00138 |
| 2774.9 | 0.00136 |
| 2773.936 | 0.00137 |
| 2772.972 | 0.00142 |
| 2772.007 | 0.00147 |
| 2771.043 | 0.00153 |
| 2770.079 | 0.00161 |
| 2769.114 | 0.00171 |
| 2768.15 | 0.0018 |
| 2767.186 | 0.0019 |
| 2766.222 | 0.00196 |
| 2765.257 | 0.00202 |
| 2764.293 | 0.00207 |
| 2763.329 | 0.00212 |
| 2762.365 | 0.00214 |
| 2761.4 | 0.00214 |
| 2760.436 | 0.00213 |
| 2759.472 | 0.00206 |
| 2758.508 | 0.00196 |
| 2757.543 | 0.00188 |
| 2756.579 | 0.00179 |
| 2755.615 | 0.00167 |
| 2754.65 | 0.00153 |
| 2753.686 | 0.00136 |
| 2752.722 | 0.00119 |
| 2751.758 | 0.0011 |
| 2750.793 | 0.00105 |
| 2749.829 | 0.00098 |
| 2748.865 | 0.00097 |
| 2747.901 | 0.00097 |
| 2746.936 | 0.001 |
| 2745.972 | 0.00107 |
| 2745.008 | 0.00117 |
| 2744.043 | 0.00129 |
| 2743.079 | 0.00144 |
| 2742.115 | 0.0016 |
| 2741.151 | 0.00172 |
| 2740.186 | 0.00181 |
| 2739.222 | 0.00187 |
| 2738.258 | 0.00192 |
| 2737.294 | 0.00193 |
| 2736.329 | 0.00186 |
| 2735.365 | 0.00178 |
| 2734.401 | 0.00172 |
| 2733.437 | 0.00166 |
| 2732.472 | 0.00153 |
| 2731.508 | 0.00143 |
| 2730.544 | 0.00134 |
| 2729.579 | 0.00127 |
| 2728.615 | 0.00117 |
| 2727.651 | 0.00104 |
| 2726.687 | 0.00095 |
| 2725.722 | 0.0009 |
| 2724.758 | 0.00088 |
| 2723.794 | 0.00094 |
| 2722.83 | 0.00107 |
| 2721.865 | 0.00119 |
| 2720.901 | 0.00131 |
| 2719.937 | 0.00141 |
| 2718.973 | 0.00144 |
| 2718.008 | 0.00143 |
| 2717.044 | 0.00147 |
| 2716.08 | 0.0015 |
| 2715.115 | 0.00145 |
| 2714.151 | 0.00136 |
| 2713.187 | 0.00127 |
| 2712.223 | 0.00126 |
| 2711.258 | 0.00128 |
| 2710.294 | 0.00129 |
| 2709.33 | 0.00132 |
| 2708.366 | 0.0014 |
| 2707.401 | 0.0015 |
| 2706.437 | 0.00158 |
| 2705.473 | 0.00164 |
| 2704.509 | 0.00175 |
| 2703.544 | 0.00183 |
| 2702.58 | 0.00184 |
| 2701.616 | 0.00189 |
| 2700.651 | 0.00197 |
| 2699.687 | 0.00199 |
| 2698.723 | 0.00196 |
| 2697.759 | 0.00188 |
| 2696.794 | 0.00178 |
| 2695.83 | 0.0017 |
| 2694.866 | 0.00163 |
| 2693.902 | 0.00156 |
| 2692.937 | 0.00142 |
| 2691.973 | 0.00124 |
| 2691.009 | 0.00111 |
| 2690.044 | 0.00106 |
| 2689.08 | 0.00105 |
| 2688.116 | 0.00104 |
| 2687.152 | 0.00108 |
| 2686.187 | 0.00117 |
| 2685.223 | 0.00124 |
| 2684.259 | 0.00126 |
| 2683.295 | 0.00126 |
| 2682.33 | 0.00124 |
| 2681.366 | 0.00123 |
| 2680.402 | 0.00125 |
| 2679.438 | 0.0013 |
| 2678.473 | 0.00141 |
| 2677.509 | 0.0015 |
| 2676.545 | 0.0015 |
| 2675.58 | 0.00147 |
| 2674.616 | 0.00142 |
| 2673.652 | 0.00138 |
| 2672.688 | 0.00135 |
| 2671.723 | 0.00133 |
| 2670.759 | 0.00131 |
| 2669.795 | 0.00121 |
| 2668.831 | 0.00108 |
| 2667.866 | 0.00098 |
| 2666.902 | 0.00097 |
| 2665.938 | 0.00102 |
| 2664.974 | 0.001 |
| 2664.009 | 0.00097 |
| 2663.045 | 0.00099 |
| 2662.081 | 0.001 |
| 2661.116 | 0.001 |
| 2660.152 | 0.001 |
| 2659.188 | 0.00104 |
| 2658.224 | 0.00114 |
| 2657.259 | 0.00132 |
| 2656.295 | 0.00156 |
| 2655.331 | 0.00179 |
| 2654.367 | 0.00195 |
| 2653.402 | 0.002 |
| 2652.438 | 0.00199 |
| 2651.474 | 0.002 |
| 2650.509 | 0.00206 |
| 2649.545 | 0.00216 |
| 2648.581 | 0.00226 |
| 2647.617 | 0.00223 |
| 2646.652 | 0.0021 |
| 2645.688 | 0.00203 |
| 2644.724 | 0.00204 |
| 2643.76 | 0.00204 |
| 2642.795 | 0.00197 |
| 2641.831 | 0.00181 |
| 2640.867 | 0.00171 |
| 2639.903 | 0.00169 |
| 2638.938 | 0.00166 |
| 2637.974 | 0.00159 |
| 2637.01 | 0.00152 |
| 2636.045 | 0.00146 |
| 2635.081 | 0.00141 |
| 2634.117 | 0.00141 |
| 2633.153 | 0.00143 |
| 2632.188 | 0.00139 |
| 2631.224 | 0.00128 |
| 2630.26 | 0.00118 |
| 2629.296 | 0.00114 |
| 2628.331 | 0.0012 |
| 2627.367 | 0.0013 |
| 2626.403 | 0.00138 |
| 2625.439 | 0.0014 |
| 2624.474 | 0.00134 |
| 2623.51 | 0.00132 |
| 2622.546 | 0.00135 |
| 2621.581 | 0.00137 |
| 2620.617 | 0.00134 |
| 2619.653 | 0.00135 |
| 2618.689 | 0.00142 |
| 2617.724 | 0.00153 |
| 2616.76 | 0.00165 |
| 2615.796 | 0.00176 |
| 2614.832 | 0.00177 |
| 2613.867 | 0.00176 |
| 2612.903 | 0.00174 |
| 2611.939 | 0.00169 |
| 2610.975 | 0.00163 |
| 2610.01 | 0.00158 |
| 2609.046 | 0.00152 |
| 2608.082 | 0.00147 |
| 2607.117 | 0.00146 |
| 2606.153 | 0.00147 |
| 2605.189 | 0.00147 |
| 2604.225 | 0.00143 |
| 2603.26 | 0.00136 |
| 2602.296 | 0.0013 |
| 2601.332 | 0.00123 |
| 2600.368 | 0.00118 |
| 2599.403 | 0.00114 |
| 2598.439 | 0.00111 |
| 2597.475 | 0.00104 |
| 2596.51 | 0.00098 |
| 2595.546 | 0.00095 |
| 2594.582 | 0.00094 |
| 2593.618 | 0.00097 |
| 2592.653 | 0.00098 |
| 2591.689 | 0.00096 |
| 2590.725 | 0.00103 |
| 2589.761 | 0.00107 |
| 2588.796 | 0.00105 |
| 2587.832 | 0.00105 |
| 2586.868 | 0.0011 |
| 2585.904 | 0.00117 |
| 2584.939 | 0.00123 |
| 2583.975 | 0.00129 |
| 2583.011 | 0.00139 |
| 2582.046 | 0.0015 |
| 2581.082 | 0.00157 |
| 2580.118 | 0.00164 |
| 2579.154 | 0.00169 |
| 2578.189 | 0.00174 |
| 2577.225 | 0.00178 |
| 2576.261 | 0.00181 |
| 2575.297 | 0.0018 |
| 2574.332 | 0.00177 |
| 2573.368 | 0.00176 |
| 2572.404 | 0.0017 |
| 2571.44 | 0.00166 |
| 2570.475 | 0.00163 |
| 2569.511 | 0.00153 |
| 2568.547 | 0.00146 |
| 2567.582 | 0.00144 |
| 2566.618 | 0.00137 |
| 2565.654 | 0.00129 |
| 2564.69 | 0.00129 |
| 2563.725 | 0.00131 |
| 2562.761 | 0.00131 |
| 2561.797 | 0.00126 |
| 2560.833 | 0.00127 |
| 2559.868 | 0.00133 |
| 2558.904 | 0.00141 |
| 2557.94 | 0.00147 |
| 2556.976 | 0.00153 |
| 2556.011 | 0.0016 |
| 2555.047 | 0.00167 |
| 2554.083 | 0.00171 |
| 2553.118 | 0.00172 |
| 2552.154 | 0.00167 |
| 2551.19 | 0.00161 |
| 2550.226 | 0.00154 |
| 2549.261 | 0.00143 |
| 2548.297 | 0.00137 |
| 2547.333 | 0.00134 |
| 2546.369 | 0.00129 |
| 2545.404 | 0.00129 |
| 2544.44 | 0.00131 |
| 2543.476 | 0.00128 |
| 2542.511 | 0.00122 |
| 2541.547 | 0.00118 |
| 2540.583 | 0.00114 |
| 2539.619 | 0.00109 |
| 2538.654 | 0.00106 |
| 2537.69 | 0.0011 |
| 2536.726 | 0.00117 |
| 2535.762 | 0.00123 |
| 2534.797 | 0.00129 |
| 2533.833 | 0.00132 |
| 2532.869 | 0.00129 |
| 2531.905 | 0.00127 |
| 2530.94 | 0.00127 |
| 2529.976 | 0.00127 |
| 2529.012 | 0.00122 |
| 2528.047 | 0.00118 |
| 2527.083 | 0.00123 |
| 2526.119 | 0.00134 |
| 2525.155 | 0.0014 |
| 2524.19 | 0.00147 |
| 2523.226 | 0.00152 |
| 2522.262 | 0.00152 |
| 2521.298 | 0.00151 |
| 2520.333 | 0.00147 |
| 2519.369 | 0.00145 |
| 2518.405 | 0.00147 |
| 2517.441 | 0.00154 |
| 2516.476 | 0.00159 |
| 2515.512 | 0.00161 |
| 2514.548 | 0.00164 |
| 2513.583 | 0.00163 |
| 2512.619 | 0.00163 |
| 2511.655 | 0.00163 |
| 2510.691 | 0.00157 |
| 2509.726 | 0.00152 |
| 2508.762 | 0.00155 |
| 2507.798 | 0.00153 |
| 2506.834 | 0.00144 |
| 2505.869 | 0.0013 |
| 2504.905 | 0.00119 |
| 2503.941 | 0.00113 |
| 2502.977 | 0.00106 |
| 2502.012 | 0.00102 |
| 2501.048 | 0.00112 |
| 2500.084 | 0.00126 |
| 2499.119 | 0.00132 |
| 2498.155 | 0.00141 |
| 2497.191 | 0.00156 |
| 2496.227 | 0.00172 |
| 2495.262 | 0.00184 |
| 2494.298 | 0.00192 |
| 2493.334 | 0.00195 |
| 2492.37 | 0.00198 |
| 2491.405 | 0.00205 |
| 2490.441 | 0.00209 |
| 2489.477 | 0.00212 |
| 2488.512 | 0.00214 |
| 2487.548 | 0.00216 |
| 2486.584 | 0.00217 |
| 2485.62 | 0.00217 |
| 2484.655 | 0.00209 |
| 2483.691 | 0.00195 |
| 2482.727 | 0.00182 |
| 2481.763 | 0.00171 |
| 2480.798 | 0.00164 |
| 2479.834 | 0.00159 |
| 2478.87 | 0.00159 |
| 2477.906 | 0.00161 |
| 2476.941 | 0.00156 |
| 2475.977 | 0.00147 |
| 2475.013 | 0.00145 |
| 2474.048 | 0.00149 |
| 2473.084 | 0.0015 |
| 2472.12 | 0.00149 |
| 2471.156 | 0.00147 |
| 2470.191 | 0.00142 |
| 2469.227 | 0.0014 |
| 2468.263 | 0.00144 |
| 2467.299 | 0.00147 |
| 2466.334 | 0.00148 |
| 2465.37 | 0.00152 |
| 2464.406 | 0.00157 |
| 2463.442 | 0.00158 |
| 2462.477 | 0.00159 |
| 2461.513 | 0.00158 |
| 2460.549 | 0.00154 |
| 2459.584 | 0.00148 |
| 2458.62 | 0.00141 |
| 2457.656 | 0.0013 |
| 2456.692 | 0.00116 |
| 2455.727 | 0.00104 |
| 2454.763 | 0.00094 |
| 2453.799 | 0.00088 |
| 2452.835 | 0.0009 |
| 2451.87 | 0.00098 |
| 2450.906 | 0.00105 |
| 2449.942 | 0.00107 |
| 2448.977 | 0.00101 |
| 2448.013 | 0.00094 |
| 2447.049 | 0.00093 |
| 2446.085 | 0.00101 |
| 2445.12 | 0.00105 |
| 2444.156 | 0.00106 |
| 2443.192 | 0.00106 |
| 2442.228 | 0.00109 |
| 2441.263 | 0.00116 |
| 2440.299 | 0.00122 |
| 2439.335 | 0.00128 |
| 2438.371 | 0.00136 |
| 2437.406 | 0.00138 |
| 2436.442 | 0.00135 |
| 2435.478 | 0.00135 |
| 2434.513 | 0.00136 |
| 2433.549 | 0.00135 |
| 2432.585 | 0.00132 |
| 2431.621 | 0.00129 |
| 2430.656 | 0.00128 |
| 2429.692 | 0.0013 |
| 2428.728 | 0.00134 |
| 2427.764 | 0.00136 |
| 2426.799 | 0.00135 |
| 2425.835 | 0.00131 |
| 2424.871 | 0.00132 |
| 2423.907 | 0.00138 |
| 2422.942 | 0.0014 |
| 2421.978 | 0.00134 |
| 2421.014 | 0.00133 |
| 2420.049 | 0.00132 |
| 2419.085 | 0.00134 |
| 2418.121 | 0.00141 |
| 2417.157 | 0.00146 |
| 2416.192 | 0.00147 |
| 2415.228 | 0.00144 |
| 2414.264 | 0.00142 |
| 2413.3 | 0.00144 |
| 2412.335 | 0.00148 |
| 2411.371 | 0.00149 |
| 2410.407 | 0.00149 |
| 2409.443 | 0.00149 |
| 2408.478 | 0.00151 |
| 2407.514 | 0.00154 |
| 2406.55 | 0.00157 |
| 2405.585 | 0.00159 |
| 2404.621 | 0.00158 |
| 2403.657 | 0.00157 |
| 2402.693 | 0.00156 |
| 2401.728 | 0.00155 |
| 2400.764 | 0.00151 |
| 2399.8 | 0.00143 |
| 2398.836 | 0.00135 |
| 2397.871 | 0.00128 |
| 2396.907 | 0.00124 |
| 2395.943 | 0.00127 |
| 2394.978 | 0.00132 |
| 2394.014 | 0.00134 |
| 2393.05 | 0.00133 |
| 2392.086 | 0.00135 |
| 2391.121 | 0.00143 |
| 2390.157 | 0.00152 |
| 2389.193 | 0.00158 |
| 2388.229 | 0.00161 |
| 2387.264 | 0.0016 |
| 2386.3 | 0.00154 |
| 2385.336 | 0.00146 |
| 2384.372 | 0.00141 |
| 2383.407 | 0.00138 |
| 2382.443 | 0.00136 |
| 2381.479 | 0.00134 |
| 2380.514 | 0.00133 |
| 2379.55 | 0.0013 |
| 2378.586 | 0.00124 |
| 2377.622 | 0.00122 |
| 2376.657 | 0.00123 |
| 2375.693 | 0.00128 |
| 2374.729 | 0.00133 |
| 2373.765 | 0.00137 |
| 2372.8 | 0.0014 |
| 2371.836 | 0.00141 |
| 2370.872 | 0.00139 |
| 2369.908 | 0.00136 |
| 2368.943 | 0.00136 |
| 2367.979 | 0.0014 |
| 2367.015 | 0.00143 |
| 2366.05 | 0.00142 |
| 2365.086 | 0.00138 |
| 2364.122 | 0.00131 |
| 2363.158 | 0.00126 |
| 2362.193 | 0.00125 |
| 2361.229 | 0.00123 |
| 2360.265 | 0.00117 |
| 2359.301 | 0.00108 |
| 2358.336 | 0.001 |
| 2357.372 | 0.00097 |
| 2356.408 | 0.00099 |
| 2355.444 | 0.00102 |
| 2354.479 | 0.00102 |
| 2353.515 | 0.00099 |
| 2352.551 | 0.00094 |
| 2351.586 | 0.00081 |
| 2350.622 | 0.00064 |
| 2349.658 | 0.0005 |
| 2348.694 | 0.00045 |
| 2347.729 | 0.00046 |
| 2346.765 | 0.0005 |
| 2345.801 | 0.00057 |
| 2344.837 | 0.00067 |
| 2343.872 | 0.00078 |
| 2342.908 | 0.00086 |
| 2341.944 | 0.00092 |
| 2340.979 | 0.00096 |
| 2340.015 | 0.00102 |
| 2339.051 | 0.00113 |
| 2338.087 | 0.00128 |
| 2337.122 | 0.00144 |
| 2336.158 | 0.00164 |
| 2335.194 | 0.00182 |
| 2334.23 | 0.00196 |
| 2333.265 | 0.00206 |
| 2332.301 | 0.0021 |
| 2331.337 | 0.00212 |
| 2330.373 | 0.00206 |
| 2329.408 | 0.00192 |
| 2328.444 | 0.00175 |
| 2327.48 | 0.00162 |
| 2326.515 | 0.00158 |
| 2325.551 | 0.00155 |
| 2324.587 | 0.0015 |
| 2323.623 | 0.00137 |
| 2322.658 | 0.00123 |
| 2321.694 | 0.00111 |
| 2320.73 | 0.00101 |
| 2319.766 | 0.00094 |
| 2318.801 | 0.00084 |
| 2317.837 | 0.00075 |
| 2316.873 | 0.00075 |
| 2315.909 | 0.00084 |
| 2314.944 | 0.0009 |
| 2313.98 | 0.00095 |
| 2313.016 | 0.001 |
| 2312.051 | 0.001 |
| 2311.087 | 0.00099 |
| 2310.123 | 0.001 |
| 2309.159 | 0.00098 |
| 2308.194 | 0.00089 |
| 2307.23 | 0.00075 |
| 2306.266 | 0.00067 |
| 2305.302 | 0.00067 |
| 2304.337 | 0.00069 |
| 2303.373 | 0.0007 |
| 2302.409 | 0.0007 |
| 2301.444 | 0.00073 |
| 2300.48 | 0.00072 |
| 2299.516 | 0.00071 |
| 2298.552 | 0.00071 |
| 2297.587 | 0.00075 |
| 2296.623 | 0.00079 |
| 2295.659 | 0.0008 |
| 2294.695 | 0.0008 |
| 2293.73 | 0.00082 |
| 2292.766 | 0.00086 |
| 2291.802 | 0.00091 |
| 2290.838 | 0.00092 |
| 2289.873 | 0.00089 |
| 2288.909 | 0.00084 |
| 2287.945 | 0.00087 |
| 2286.98 | 0.00095 |
| 2286.016 | 0.00103 |
| 2285.052 | 0.00112 |
| 2284.088 | 0.00118 |
| 2283.123 | 0.00127 |
| 2282.159 | 0.00136 |
| 2281.195 | 0.00142 |
| 2280.231 | 0.00148 |
| 2279.266 | 0.00158 |
| 2278.302 | 0.00167 |
| 2277.338 | 0.00169 |
| 2276.374 | 0.00169 |
| 2275.409 | 0.00173 |
| 2274.445 | 0.00181 |
| 2273.481 | 0.00186 |
| 2272.516 | 0.00183 |
| 2271.552 | 0.00174 |
| 2270.588 | 0.00164 |
| 2269.624 | 0.00154 |
| 2268.659 | 0.00141 |
| 2267.695 | 0.00123 |
| 2266.731 | 0.00108 |
| 2265.767 | 0.00103 |
| 2264.802 | 0.00108 |
| 2263.838 | 0.00113 |
| 2262.874 | 0.00115 |
| 2261.91 | 0.00114 |
| 2260.945 | 0.0011 |
| 2259.981 | 0.00104 |
| 2259.017 | 0.00097 |
| 2258.052 | 0.00091 |
| 2257.088 | 0.00091 |
| 2256.124 | 0.00095 |
| 2255.16 | 0.001 |
| 2254.195 | 0.00102 |
| 2253.231 | 0.00103 |
| 2252.267 | 0.00105 |
| 2251.303 | 0.00109 |
| 2250.338 | 0.0011 |
| 2249.374 | 0.00107 |
| 2248.41 | 0.00105 |
| 2247.445 | 0.00108 |
| 2246.481 | 0.00112 |
| 2245.517 | 0.00119 |
| 2244.553 | 0.00127 |
| 2243.588 | 0.00134 |
| 2242.624 | 0.00143 |
| 2241.66 | 0.00153 |
| 2240.696 | 0.00154 |
| 2239.731 | 0.00145 |
| 2238.767 | 0.00141 |
| 2237.803 | 0.00141 |
| 2236.839 | 0.00142 |
| 2235.874 | 0.00145 |
| 2234.91 | 0.00149 |
| 2233.946 | 0.00157 |
| 2232.981 | 0.00164 |
| 2232.017 | 0.00165 |
| 2231.053 | 0.00159 |
| 2230.089 | 0.00153 |
| 2229.124 | 0.00151 |
| 2228.16 | 0.00151 |
| 2227.196 | 0.00149 |
| 2226.232 | 0.00146 |
| 2225.267 | 0.00147 |
| 2224.303 | 0.00152 |
| 2223.339 | 0.00157 |
| 2222.375 | 0.00162 |
| 2221.41 | 0.00161 |
| 2220.446 | 0.00155 |
| 2219.482 | 0.00149 |
| 2218.517 | 0.00141 |
| 2217.553 | 0.0013 |
| 2216.589 | 0.00124 |
| 2215.625 | 0.00124 |
| 2214.66 | 0.00127 |
| 2213.696 | 0.00125 |
| 2212.732 | 0.00121 |
| 2211.768 | 0.00122 |
| 2210.803 | 0.00131 |
| 2209.839 | 0.00139 |
| 2208.875 | 0.00138 |
| 2207.911 | 0.00136 |
| 2206.946 | 0.00139 |
| 2205.982 | 0.00145 |
| 2205.018 | 0.00154 |
| 2204.053 | 0.00158 |
| 2203.089 | 0.00164 |
| 2202.125 | 0.00172 |
| 2201.161 | 0.00178 |
| 2200.196 | 0.00182 |
| 2199.232 | 0.00182 |
| 2198.268 | 0.00179 |
| 2197.304 | 0.00178 |
| 2196.339 | 0.00173 |
| 2195.375 | 0.00167 |
| 2194.411 | 0.00167 |
| 2193.446 | 0.00167 |
| 2192.482 | 0.0016 |
| 2191.518 | 0.00151 |
| 2190.554 | 0.00148 |
| 2189.589 | 0.00154 |
| 2188.625 | 0.00167 |
| 2187.661 | 0.00176 |
| 2186.697 | 0.00175 |
| 2185.732 | 0.00171 |
| 2184.768 | 0.00168 |
| 2183.804 | 0.00168 |
| 2182.84 | 0.00171 |
| 2181.875 | 0.00172 |
| 2180.911 | 0.00167 |
| 2179.947 | 0.00164 |
| 2178.982 | 0.00158 |
| 2178.018 | 0.00152 |
| 2177.054 | 0.00146 |
| 2176.09 | 0.00141 |
| 2175.125 | 0.00139 |
| 2174.161 | 0.00135 |
| 2173.197 | 0.00124 |
| 2172.233 | 0.0012 |
| 2171.268 | 0.00126 |
| 2170.304 | 0.00134 |
| 2169.34 | 0.00133 |
| 2168.376 | 0.00134 |
| 2167.411 | 0.00142 |
| 2166.447 | 0.00151 |
| 2165.483 | 0.0015 |
| 2164.518 | 0.00142 |
| 2163.554 | 0.00136 |
| 2162.59 | 0.00134 |
| 2161.626 | 0.00134 |
| 2160.661 | 0.00132 |
| 2159.697 | 0.00138 |
| 2158.733 | 0.00143 |
| 2157.769 | 0.00144 |
| 2156.804 | 0.00144 |
| 2155.84 | 0.00147 |
| 2154.876 | 0.00152 |
| 2153.911 | 0.00158 |
| 2152.947 | 0.00166 |
| 2151.983 | 0.00178 |
| 2151.019 | 0.00187 |
| 2150.054 | 0.00188 |
| 2149.09 | 0.00192 |
| 2148.126 | 0.00203 |
| 2147.162 | 0.00214 |
| 2146.197 | 0.00212 |
| 2145.233 | 0.00206 |
| 2144.269 | 0.002 |
| 2143.305 | 0.00194 |
| 2142.34 | 0.00182 |
| 2141.376 | 0.00174 |
| 2140.412 | 0.00174 |
| 2139.447 | 0.00179 |
| 2138.483 | 0.00177 |
| 2137.519 | 0.00168 |
| 2136.555 | 0.00164 |
| 2135.59 | 0.00157 |
| 2134.626 | 0.00147 |
| 2133.662 | 0.0014 |
| 2132.698 | 0.00131 |
| 2131.733 | 0.00123 |
| 2130.769 | 0.0012 |
| 2129.805 | 0.0012 |
| 2128.841 | 0.00119 |
| 2127.876 | 0.00112 |
| 2126.912 | 0.00101 |
| 2125.948 | 0.00099 |
| 2124.983 | 0.00103 |
| 2124.019 | 0.00108 |
| 2123.055 | 0.00109 |
| 2122.091 | 0.0011 |
| 2121.126 | 0.00117 |
| 2120.162 | 0.00128 |
| 2119.198 | 0.00134 |
| 2118.234 | 0.00139 |
| 2117.269 | 0.00148 |
| 2116.305 | 0.00158 |
| 2115.341 | 0.00155 |
| 2114.377 | 0.00151 |
| 2113.412 | 0.00153 |
| 2112.448 | 0.00155 |
| 2111.484 | 0.00154 |
| 2110.519 | 0.00151 |
| 2109.555 | 0.00145 |
| 2108.591 | 0.00142 |
| 2107.627 | 0.00143 |
| 2106.662 | 0.0014 |
| 2105.698 | 0.00135 |
| 2104.734 | 0.00129 |
| 2103.77 | 0.00126 |
| 2102.805 | 0.00127 |
| 2101.841 | 0.00132 |
| 2100.877 | 0.00138 |
| 2099.912 | 0.00138 |
| 2098.948 | 0.00133 |
| 2097.984 | 0.00125 |
| 2097.02 | 0.00118 |
| 2096.055 | 0.00114 |
| 2095.091 | 0.00118 |
| 2094.127 | 0.00126 |
| 2093.163 | 0.00127 |
| 2092.198 | 0.00125 |
| 2091.234 | 0.00133 |
| 2090.27 | 0.00144 |
| 2089.306 | 0.00152 |
| 2088.341 | 0.00159 |
| 2087.377 | 0.00165 |
| 2086.413 | 0.00169 |
| 2085.448 | 0.00171 |
| 2084.484 | 0.00171 |
| 2083.52 | 0.00167 |
| 2082.556 | 0.00162 |
| 2081.591 | 0.00157 |
| 2080.627 | 0.00153 |
| 2079.663 | 0.00147 |
| 2078.699 | 0.00141 |
| 2077.734 | 0.00133 |
| 2076.77 | 0.00126 |
| 2075.806 | 0.0012 |
| 2074.842 | 0.00118 |
| 2073.877 | 0.00118 |
| 2072.913 | 0.00118 |
| 2071.949 | 0.00116 |
| 2070.984 | 0.00111 |
| 2070.02 | 0.00108 |
| 2069.056 | 0.00113 |
| 2068.092 | 0.00123 |
| 2067.127 | 0.00125 |
| 2066.163 | 0.00127 |
| 2065.199 | 0.00131 |
| 2064.235 | 0.00134 |
| 2063.27 | 0.00139 |
| 2062.306 | 0.00146 |
| 2061.342 | 0.00154 |
| 2060.378 | 0.00157 |
| 2059.413 | 0.00155 |
| 2058.449 | 0.0015 |
| 2057.485 | 0.00144 |
| 2056.52 | 0.00143 |
| 2055.556 | 0.00149 |
| 2054.592 | 0.00156 |
| 2053.628 | 0.00158 |
| 2052.663 | 0.00155 |
| 2051.699 | 0.00145 |
| 2050.735 | 0.00131 |
| 2049.771 | 0.0012 |
| 2048.806 | 0.00113 |
| 2047.842 | 0.00109 |
| 2046.878 | 0.00105 |
| 2045.913 | 0.00101 |
| 2044.949 | 0.00096 |
| 2043.985 | 0.00087 |
| 2043.021 | 0.00084 |
| 2042.056 | 0.00083 |
| 2041.092 | 0.00083 |
| 2040.128 | 0.00088 |
| 2039.164 | 0.00096 |
| 2038.199 | 0.00107 |
| 2037.235 | 0.00116 |
| 2036.271 | 0.00115 |
| 2035.307 | 0.00112 |
| 2034.342 | 0.00116 |
| 2033.378 | 0.00131 |
| 2032.414 | 0.00146 |
| 2031.449 | 0.00158 |
| 2030.485 | 0.00166 |
| 2029.521 | 0.00171 |
| 2028.557 | 0.00173 |
| 2027.592 | 0.00177 |
| 2026.628 | 0.00181 |
| 2025.664 | 0.00184 |
| 2024.7 | 0.00187 |
| 2023.735 | 0.00189 |
| 2022.771 | 0.00191 |
| 2021.807 | 0.00188 |
| 2020.843 | 0.00182 |
| 2019.878 | 0.00177 |
| 2018.914 | 0.00171 |
| 2017.95 | 0.00166 |
| 2016.985 | 0.00161 |
| 2016.021 | 0.00158 |
| 2015.057 | 0.00152 |
| 2014.093 | 0.0014 |
| 2013.128 | 0.00125 |
| 2012.164 | 0.00123 |
| 2011.2 | 0.00131 |
| 2010.236 | 0.00138 |
| 2009.271 | 0.00144 |
| 2008.307 | 0.00143 |
| 2007.343 | 0.00136 |
| 2006.379 | 0.00128 |
| 2005.414 | 0.00121 |
| 2004.45 | 0.00117 |
| 2003.486 | 0.00116 |
| 2002.521 | 0.00119 |
| 2001.557 | 0.00122 |
| 2000.593 | 0.00122 |
| 1999.629 | 0.00122 |
| 1998.664 | 0.00126 |
| 1997.7 | 0.00135 |
| 1996.736 | 0.00142 |
| 1995.772 | 0.00147 |
| 1994.807 | 0.00148 |
| 1993.843 | 0.00149 |
| 1992.879 | 0.00148 |
| 1991.914 | 0.00144 |
| 1990.95 | 0.00145 |
| 1989.986 | 0.00155 |
| 1989.022 | 0.00165 |
| 1988.057 | 0.0017 |
| 1987.093 | 0.0017 |
| 1986.129 | 0.00172 |
| 1985.165 | 0.00171 |
| 1984.2 | 0.00174 |
| 1983.236 | 0.00175 |
| 1982.272 | 0.00174 |
| 1981.308 | 0.00171 |
| 1980.343 | 0.00169 |
| 1979.379 | 0.00164 |
| 1978.415 | 0.00156 |
| 1977.45 | 0.0015 |
| 1976.486 | 0.00151 |
| 1975.522 | 0.00162 |
| 1974.558 | 0.00176 |
| 1973.593 | 0.00182 |
| 1972.629 | 0.00178 |
| 1971.665 | 0.00173 |
| 1970.701 | 0.00172 |
| 1969.736 | 0.0017 |
| 1968.772 | 0.00175 |
| 1967.808 | 0.00181 |
| 1966.844 | 0.00183 |
| 1965.879 | 0.00183 |
| 1964.915 | 0.00183 |
| 1963.951 | 0.00182 |
| 1962.986 | 0.00185 |
| 1962.022 | 0.0019 |
| 1961.058 | 0.00192 |
| 1960.094 | 0.00189 |
| 1959.129 | 0.00183 |
| 1958.165 | 0.00179 |
| 1957.201 | 0.0017 |
| 1956.237 | 0.00159 |
| 1955.272 | 0.00149 |
| 1954.308 | 0.00144 |
| 1953.344 | 0.00145 |
| 1952.379 | 0.0015 |
| 1951.415 | 0.00151 |
| 1950.451 | 0.00142 |
| 1949.487 | 0.00131 |
| 1948.522 | 0.0013 |
| 1947.558 | 0.00133 |
| 1946.594 | 0.00129 |
| 1945.63 | 0.00121 |
| 1944.665 | 0.00111 |
| 1943.701 | 0.00107 |
| 1942.737 | 0.00107 |
| 1941.773 | 0.00109 |
| 1940.808 | 0.00112 |
| 1939.844 | 0.0012 |
| 1938.88 | 0.00126 |
| 1937.915 | 0.00129 |
| 1936.951 | 0.00128 |
| 1935.987 | 0.00124 |
| 1935.023 | 0.0012 |
| 1934.058 | 0.00116 |
| 1933.094 | 0.00115 |
| 1932.13 | 0.00116 |
| 1931.166 | 0.00115 |
| 1930.201 | 0.00116 |
| 1929.237 | 0.00114 |
| 1928.273 | 0.0011 |
| 1927.309 | 0.00106 |
| 1926.344 | 0.00102 |
| 1925.38 | 0.00097 |
| 1924.416 | 0.0009 |
| 1923.451 | 0.00085 |
| 1922.487 | 0.00088 |
| 1921.523 | 0.0009 |
| 1920.559 | 0.00096 |
| 1919.594 | 0.00105 |
| 1918.63 | 0.00113 |
| 1917.666 | 0.00117 |
| 1916.702 | 0.00121 |
| 1915.737 | 0.00121 |
| 1914.773 | 0.0012 |
| 1913.809 | 0.0012 |
| 1912.845 | 0.00123 |
| 1911.88 | 0.00133 |
| 1910.916 | 0.00143 |
| 1909.952 | 0.00145 |
| 1908.987 | 0.00142 |
| 1908.023 | 0.0014 |
| 1907.059 | 0.00143 |
| 1906.095 | 0.00144 |
| 1905.13 | 0.00142 |
| 1904.166 | 0.00142 |
| 1903.202 | 0.00145 |
| 1902.238 | 0.00145 |
| 1901.273 | 0.00144 |
| 1900.309 | 0.00141 |
| 1899.345 | 0.00136 |
| 1898.38 | 0.00132 |
| 1897.416 | 0.00135 |
| 1896.452 | 0.00143 |
| 1895.488 | 0.00143 |
| 1894.523 | 0.00139 |
| 1893.559 | 0.00137 |
| 1892.595 | 0.00139 |
| 1891.631 | 0.00145 |
| 1890.666 | 0.00154 |
| 1889.702 | 0.00159 |
| 1888.738 | 0.00158 |
| 1887.774 | 0.00153 |
| 1886.809 | 0.00144 |
| 1885.845 | 0.0014 |
| 1884.881 | 0.00142 |
| 1883.916 | 0.00146 |
| 1882.952 | 0.00142 |
| 1881.988 | 0.00136 |
| 1881.024 | 0.00132 |
| 1880.059 | 0.0013 |
| 1879.095 | 0.00129 |
| 1878.131 | 0.00125 |
| 1877.167 | 0.00119 |
| 1876.202 | 0.00118 |
| 1875.238 | 0.00117 |
| 1874.274 | 0.00114 |
| 1873.31 | 0.00116 |
| 1872.345 | 0.0012 |
| 1871.381 | 0.00125 |
| 1870.417 | 0.00133 |
| 1869.452 | 0.0014 |
| 1868.488 | 0.00142 |
| 1867.524 | 0.00137 |
| 1866.56 | 0.00129 |
| 1865.595 | 0.00125 |
| 1864.631 | 0.00124 |
| 1863.667 | 0.00126 |
| 1862.703 | 0.00127 |
| 1861.738 | 0.00125 |
| 1860.774 | 0.00127 |
| 1859.81 | 0.00132 |
| 1858.846 | 0.00138 |
| 1857.881 | 0.00143 |
| 1856.917 | 0.00148 |
| 1855.953 | 0.00149 |
| 1854.988 | 0.00145 |
| 1854.024 | 0.00138 |
| 1853.06 | 0.00133 |
| 1852.096 | 0.00132 |
| 1851.131 | 0.00131 |
| 1850.167 | 0.00129 |
| 1849.203 | 0.00125 |
| 1848.239 | 0.00122 |
| 1847.274 | 0.00117 |
| 1846.31 | 0.00109 |
| 1845.346 | 0.00102 |
| 1844.381 | 0.00094 |
| 1843.417 | 0.00084 |
| 1842.453 | 0.00075 |
| 1841.489 | 0.0007 |
| 1840.524 | 0.00068 |
| 1839.56 | 0.00067 |
| 1838.596 | 0.00075 |
| 1837.632 | 0.00092 |
| 1836.667 | 0.00105 |
| 1835.703 | 0.00111 |
| 1834.739 | 0.00115 |
| 1833.775 | 0.00112 |
| 1832.81 | 0.0011 |
| 1831.846 | 0.00115 |
| 1830.882 | 0.00121 |
| 1829.917 | 0.00129 |
| 1828.953 | 0.00139 |
| 1827.989 | 0.00144 |
| 1827.025 | 0.00148 |
| 1826.06 | 0.0016 |
| 1825.096 | 0.00174 |
| 1824.132 | 0.00184 |
| 1823.168 | 0.00192 |
| 1822.203 | 0.00196 |
| 1821.239 | 0.00196 |
| 1820.275 | 0.00191 |
| 1819.311 | 0.00189 |
| 1818.346 | 0.00187 |
| 1817.382 | 0.00183 |
| 1816.418 | 0.00181 |
| 1815.453 | 0.00184 |
| 1814.489 | 0.00184 |
| 1813.525 | 0.00173 |
| 1812.561 | 0.0016 |
| 1811.596 | 0.00147 |
| 1810.632 | 0.00131 |
| 1809.668 | 0.00122 |
| 1808.704 | 0.00118 |
| 1807.739 | 0.00116 |
| 1806.775 | 0.00115 |
| 1805.811 | 0.00117 |
| 1804.846 | 0.00122 |
| 1803.882 | 0.00123 |
| 1802.918 | 0.00126 |
| 1801.954 | 0.00133 |
| 1800.989 | 0.00141 |
| 1800.025 | 0.00148 |
| 1799.061 | 0.00151 |
| 1798.097 | 0.00153 |
| 1797.132 | 0.00156 |
| 1796.168 | 0.00159 |
| 1795.204 | 0.00164 |
| 1794.24 | 0.00168 |
| 1793.275 | 0.00169 |
| 1792.311 | 0.00171 |
| 1791.347 | 0.00168 |
| 1790.382 | 0.0016 |
| 1789.418 | 0.00158 |
| 1788.454 | 0.00165 |
| 1787.49 | 0.0017 |
| 1786.525 | 0.0017 |
| 1785.561 | 0.00171 |
| 1784.597 | 0.00169 |
| 1783.633 | 0.00168 |
| 1782.668 | 0.0017 |
| 1781.704 | 0.0017 |
| 1780.74 | 0.00165 |
| 1779.776 | 0.00165 |
| 1778.811 | 0.00168 |
| 1777.847 | 0.00168 |
| 1776.883 | 0.00168 |
| 1775.918 | 0.00169 |
| 1774.954 | 0.00171 |
| 1773.99 | 0.00172 |
| 1773.026 | 0.00173 |
| 1772.061 | 0.0017 |
| 1771.097 | 0.00158 |
| 1770.133 | 0.00145 |
| 1769.169 | 0.00135 |
| 1768.204 | 0.00127 |
| 1767.24 | 0.00125 |
| 1766.276 | 0.00127 |
| 1765.312 | 0.00129 |
| 1764.347 | 0.00127 |
| 1763.383 | 0.00123 |
| 1762.419 | 0.00122 |
| 1761.454 | 0.00119 |
| 1760.49 | 0.00117 |
| 1759.526 | 0.00115 |
| 1758.562 | 0.00109 |
| 1757.597 | 0.00104 |
| 1756.633 | 0.00103 |
| 1755.669 | 0.00106 |
| 1754.705 | 0.00112 |
| 1753.74 | 0.00118 |
| 1752.776 | 0.00123 |
| 1751.812 | 0.00127 |
| 1750.847 | 0.00131 |
| 1749.883 | 0.00138 |
| 1748.919 | 0.00141 |
| 1747.955 | 0.00144 |
| 1746.99 | 0.00145 |
| 1746.026 | 0.00147 |
| 1745.062 | 0.00147 |
| 1744.098 | 0.0015 |
| 1743.133 | 0.00157 |
| 1742.169 | 0.00169 |
| 1741.205 | 0.00181 |
| 1740.241 | 0.00187 |
| 1739.276 | 0.00186 |
| 1738.312 | 0.00179 |
| 1737.348 | 0.00171 |
| 1736.383 | 0.00163 |
| 1735.419 | 0.00155 |
| 1734.455 | 0.00145 |
| 1733.491 | 0.00137 |
| 1732.526 | 0.00132 |
| 1731.562 | 0.00129 |
| 1730.598 | 0.00123 |
| 1729.634 | 0.00116 |
| 1728.669 | 0.00111 |
| 1727.705 | 0.00108 |
| 1726.741 | 0.00105 |
| 1725.777 | 0.001 |
| 1724.812 | 0.00097 |
| 1723.848 | 0.00093 |
| 1722.884 | 0.00084 |
| 1721.919 | 0.00076 |
| 1720.955 | 0.00077 |
| 1719.991 | 0.00086 |
| 1719.027 | 0.00096 |
| 1718.062 | 0.00103 |
| 1717.098 | 0.00106 |
| 1716.134 | 0.00112 |
| 1715.17 | 0.00121 |
| 1714.205 | 0.00131 |
| 1713.241 | 0.00138 |
| 1712.277 | 0.00141 |
| 1711.313 | 0.00145 |
| 1710.348 | 0.00151 |
| 1709.384 | 0.00157 |
| 1708.42 | 0.00161 |
| 1707.455 | 0.00159 |
| 1706.491 | 0.00154 |
| 1705.527 | 0.00146 |
| 1704.563 | 0.00136 |
| 1703.598 | 0.0013 |
| 1702.634 | 0.00131 |
| 1701.67 | 0.00124 |
| 1700.706 | 0.00114 |
| 1699.741 | 0.00105 |
| 1698.777 | 0.00108 |
| 1697.813 | 0.00119 |
| 1696.848 | 0.00129 |
| 1695.884 | 0.00137 |
| 1694.92 | 0.00142 |
| 1693.956 | 0.00147 |
| 1692.991 | 0.00154 |
| 1692.027 | 0.00156 |
| 1691.063 | 0.00153 |
| 1690.099 | 0.00149 |
| 1689.134 | 0.00148 |
| 1688.17 | 0.00147 |
| 1687.206 | 0.00143 |
| 1686.242 | 0.00137 |
| 1685.277 | 0.00133 |
| 1684.313 | 0.00125 |
| 1683.349 | 0.00117 |
| 1682.384 | 0.00107 |
| 1681.42 | 0.00098 |
| 1680.456 | 0.00095 |
| 1679.492 | 0.00091 |
| 1678.527 | 0.00084 |
| 1677.563 | 0.00089 |
| 1676.599 | 0.00102 |
| 1675.635 | 0.00117 |
| 1674.67 | 0.00126 |
| 1673.706 | 0.00126 |
| 1672.742 | 0.00122 |
| 1671.778 | 0.00116 |
| 1670.813 | 0.00111 |
| 1669.849 | 0.00107 |
| 1668.885 | 0.00104 |
| 1667.92 | 0.00104 |
| 1666.956 | 0.00108 |
| 1665.992 | 0.00116 |
| 1665.028 | 0.00123 |
| 1664.063 | 0.00134 |
| 1663.099 | 0.00147 |
| 1662.135 | 0.00157 |
| 1661.171 | 0.00159 |
| 1660.206 | 0.0016 |
| 1659.242 | 0.00156 |
| 1658.278 | 0.00151 |
| 1657.313 | 0.00154 |
| 1656.349 | 0.00158 |
| 1655.385 | 0.00158 |
| 1654.421 | 0.00159 |
| 1653.456 | 0.00158 |
| 1652.492 | 0.00153 |
| 1651.528 | 0.00143 |
| 1650.564 | 0.00131 |
| 1649.599 | 0.00119 |
| 1648.635 | 0.00109 |
| 1647.671 | 0.00106 |
| 1646.707 | 0.00109 |
| 1645.742 | 0.00117 |
| 1644.778 | 0.00126 |
| 1643.814 | 0.00133 |
| 1642.849 | 0.00138 |
| 1641.885 | 0.00144 |
| 1640.921 | 0.00147 |
| 1639.957 | 0.0015 |
| 1638.992 | 0.00148 |
| 1638.028 | 0.00144 |
| 1637.064 | 0.00145 |
| 1636.1 | 0.00149 |
| 1635.135 | 0.00152 |
| 1634.171 | 0.00157 |
| 1633.207 | 0.00156 |
| 1632.243 | 0.00151 |
| 1631.278 | 0.00149 |
| 1630.314 | 0.00146 |
| 1629.35 | 0.00145 |
| 1628.385 | 0.00145 |
| 1627.421 | 0.00142 |
| 1626.457 | 0.00139 |
| 1625.493 | 0.0014 |
| 1624.528 | 0.00144 |
| 1623.564 | 0.00144 |
| 1622.6 | 0.00138 |
| 1621.636 | 0.00132 |
| 1620.671 | 0.00125 |
| 1619.707 | 0.0012 |
| 1618.743 | 0.00119 |
| 1617.779 | 0.00123 |
| 1616.814 | 0.00127 |
| 1615.85 | 0.00128 |
| 1614.886 | 0.0013 |
| 1613.921 | 0.00135 |
| 1612.957 | 0.00143 |
| 1611.993 | 0.00149 |
| 1611.029 | 0.00154 |
| 1610.064 | 0.00158 |
| 1609.1 | 0.00162 |
| 1608.136 | 0.00164 |
| 1607.172 | 0.00165 |
| 1606.207 | 0.00164 |
| 1605.243 | 0.0016 |
| 1604.279 | 0.00154 |
| 1603.314 | 0.00149 |
| 1602.35 | 0.00138 |
| 1601.386 | 0.00124 |
| 1600.422 | 0.00107 |
| 1599.457 | 0.00097 |
| 1598.493 | 0.00094 |
| 1597.529 | 0.00089 |
| 1596.565 | 0.00082 |
| 1595.6 | 0.00076 |
| 1594.636 | 0.00073 |
| 1593.672 | 0.00069 |
| 1592.708 | 0.00064 |
| 1591.743 | 0.00066 |
| 1590.779 | 0.00076 |
| 1589.815 | 0.00087 |
| 1588.85 | 0.00097 |
| 1587.886 | 0.00107 |
| 1586.922 | 0.00117 |
| 1585.958 | 0.00124 |
| 1584.993 | 0.00127 |
| 1584.029 | 0.00129 |
| 1583.065 | 0.00131 |
| 1582.101 | 0.00135 |
| 1581.136 | 0.00138 |
| 1580.172 | 0.00134 |
| 1579.208 | 0.00127 |
| 1578.244 | 0.00124 |
| 1577.279 | 0.00119 |
| 1576.315 | 0.00115 |
| 1575.351 | 0.0011 |
| 1574.386 | 0.00101 |
| 1573.422 | 0.00091 |
| 1572.458 | 0.00085 |
| 1571.494 | 0.00083 |
| 1570.529 | 0.00083 |
| 1569.565 | 0.00085 |
| 1568.601 | 0.00089 |
| 1567.637 | 0.00092 |
| 1566.672 | 0.00091 |
| 1565.708 | 0.00092 |
| 1564.744 | 0.00092 |
| 1563.78 | 0.00093 |
| 1562.815 | 0.00094 |
| 1561.851 | 0.00095 |
| 1560.887 | 0.001 |
| 1559.922 | 0.00106 |
| 1558.958 | 0.0011 |
| 1557.994 | 0.00108 |
| 1557.03 | 0.00107 |
| 1556.065 | 0.00113 |
| 1555.101 | 0.00118 |
| 1554.137 | 0.00122 |
| 1553.173 | 0.00128 |
| 1552.208 | 0.00136 |
| 1551.244 | 0.00143 |
| 1550.28 | 0.00146 |
| 1549.315 | 0.00153 |
| 1548.351 | 0.00164 |
| 1547.387 | 0.00175 |
| 1546.423 | 0.00183 |
| 1545.458 | 0.00184 |
| 1544.494 | 0.00182 |
| 1543.53 | 0.00184 |
| 1542.566 | 0.00181 |
| 1541.601 | 0.00172 |
| 1540.637 | 0.00163 |
| 1539.673 | 0.00153 |
| 1538.709 | 0.00147 |
| 1537.744 | 0.00146 |
| 1536.78 | 0.00145 |
| 1535.816 | 0.00143 |
| 1534.851 | 0.00145 |
| 1533.887 | 0.0015 |
| 1532.923 | 0.00153 |
| 1531.959 | 0.00152 |
| 1530.994 | 0.00155 |
| 1530.03 | 0.00159 |
| 1529.066 | 0.00159 |
| 1528.102 | 0.00156 |
| 1527.137 | 0.00151 |
| 1526.173 | 0.0015 |
| 1525.209 | 0.00149 |
| 1524.245 | 0.00147 |
| 1523.28 | 0.00141 |
| 1522.316 | 0.00131 |
| 1521.352 | 0.00126 |
| 1520.387 | 0.00124 |
| 1519.423 | 0.00117 |
| 1518.459 | 0.0011 |
| 1517.495 | 0.00106 |
| 1516.53 | 0.00105 |
| 1515.566 | 0.00104 |
| 1514.602 | 0.00107 |
| 1513.638 | 0.00111 |
| 1512.673 | 0.00115 |
| 1511.709 | 0.00117 |
| 1510.745 | 0.00114 |
| 1509.781 | 0.00112 |
| 1508.816 | 0.00115 |
| 1507.852 | 0.00119 |
| 1506.888 | 0.00119 |
| 1505.923 | 0.00115 |
| 1504.959 | 0.00113 |
| 1503.995 | 0.00115 |
| 1503.031 | 0.0012 |
| 1502.066 | 0.0012 |
| 1501.102 | 0.00122 |
| 1500.138 | 0.00125 |
| 1499.174 | 0.00129 |
| 1498.209 | 0.00135 |
| 1497.245 | 0.00141 |
| 1496.281 | 0.00146 |
| 1495.316 | 0.00149 |
| 1494.352 | 0.0015 |
| 1493.388 | 0.00151 |
| 1492.424 | 0.00151 |
| 1491.459 | 0.00149 |
| 1490.495 | 0.00147 |
| 1489.531 | 0.00144 |
| 1488.567 | 0.0014 |
| 1487.602 | 0.00134 |
| 1486.638 | 0.0013 |
| 1485.674 | 0.00126 |
| 1484.71 | 0.00124 |
| 1483.745 | 0.00122 |
| 1482.781 | 0.00122 |
| 1481.817 | 0.00126 |
| 1480.852 | 0.00127 |
| 1479.888 | 0.00125 |
| 1478.924 | 0.00121 |
| 1477.96 | 0.00121 |
| 1476.995 | 0.00121 |
| 1476.031 | 0.00121 |
| 1475.067 | 0.00124 |
| 1474.103 | 0.00126 |
| 1473.138 | 0.00132 |
| 1472.174 | 0.00138 |
| 1471.21 | 0.00145 |
| 1470.246 | 0.0015 |
| 1469.281 | 0.00151 |
| 1468.317 | 0.00144 |
| 1467.353 | 0.00142 |
| 1466.388 | 0.00142 |
| 1465.424 | 0.00139 |
| 1464.46 | 0.0013 |
| 1463.496 | 0.00126 |
| 1462.531 | 0.00126 |
| 1461.567 | 0.00127 |
| 1460.603 | 0.00126 |
| 1459.639 | 0.00124 |
| 1458.674 | 0.00122 |
| 1457.71 | 0.00116 |
| 1456.746 | 0.00111 |
| 1455.781 | 0.00112 |
| 1454.817 | 0.00109 |
| 1453.853 | 0.00098 |
| 1452.889 | 0.00091 |
| 1451.924 | 0.00088 |
| 1450.96 | 0.0009 |
| 1449.996 | 0.00097 |
| 1449.032 | 0.00106 |
| 1448.067 | 0.00113 |
| 1447.103 | 0.00119 |
| 1446.139 | 0.00123 |
| 1445.175 | 0.00127 |
| 1444.21 | 0.00131 |
| 1443.246 | 0.00134 |
| 1442.282 | 0.00136 |
| 1441.317 | 0.00137 |
| 1440.353 | 0.00139 |
| 1439.389 | 0.0014 |
| 1438.425 | 0.00138 |
| 1437.46 | 0.00134 |
| 1436.496 | 0.00128 |
| 1435.532 | 0.00122 |
| 1434.568 | 0.00118 |
| 1433.603 | 0.0011 |
| 1432.639 | 0.00098 |
| 1431.675 | 0.00082 |
| 1430.711 | 0.0007 |
| 1429.746 | 0.00068 |
| 1428.782 | 0.00074 |
| 1427.818 | 0.00082 |
| 1426.853 | 0.00089 |
| 1425.889 | 0.00096 |
| 1424.925 | 0.00099 |
| 1423.961 | 0.00102 |
| 1422.996 | 0.00107 |
| 1422.032 | 0.0011 |
| 1421.068 | 0.00113 |
| 1420.104 | 0.00118 |
| 1419.139 | 0.00118 |
| 1418.175 | 0.00115 |
| 1417.211 | 0.00115 |
| 1416.247 | 0.00116 |
| 1415.282 | 0.0012 |
| 1414.318 | 0.00126 |
| 1413.354 | 0.00131 |
| 1412.389 | 0.00133 |
| 1411.425 | 0.00131 |
| 1410.461 | 0.00127 |
| 1409.497 | 0.00127 |
| 1408.532 | 0.00135 |
| 1407.568 | 0.00141 |
| 1406.604 | 0.00145 |
| 1405.64 | 0.0015 |
| 1404.675 | 0.00153 |
| 1403.711 | 0.00154 |
| 1402.747 | 0.00147 |
| 1401.782 | 0.00136 |
| 1400.818 | 0.00131 |
| 1399.854 | 0.00131 |
| 1398.89 | 0.00126 |
| 1397.925 | 0.00118 |
| 1396.961 | 0.00112 |
| 1395.997 | 0.00106 |
| 1395.033 | 0.00102 |
| 1394.068 | 0.00099 |
| 1393.104 | 0.00101 |
| 1392.14 | 0.0011 |
| 1391.176 | 0.00118 |
| 1390.211 | 0.00125 |
| 1389.247 | 0.00129 |
| 1388.283 | 0.00132 |
| 1387.318 | 0.00141 |
| 1386.354 | 0.00156 |
| 1385.39 | 0.00167 |
| 1384.426 | 0.00169 |
| 1383.461 | 0.00173 |
| 1382.497 | 0.00178 |
| 1381.533 | 0.00178 |
| 1380.569 | 0.00174 |
| 1379.604 | 0.00167 |
| 1378.64 | 0.00161 |
| 1377.676 | 0.00158 |
| 1376.712 | 0.00153 |
| 1375.747 | 0.00146 |
| 1374.783 | 0.00141 |
| 1373.819 | 0.00139 |
| 1372.854 | 0.00136 |
| 1371.89 | 0.0013 |
| 1370.926 | 0.00124 |
| 1369.962 | 0.00125 |
| 1368.997 | 0.00131 |
| 1368.033 | 0.00141 |
| 1367.069 | 0.0015 |
| 1366.105 | 0.00149 |
| 1365.14 | 0.00146 |
| 1364.176 | 0.00147 |
| 1363.212 | 0.00151 |
| 1362.248 | 0.0015 |
| 1361.283 | 0.00147 |
| 1360.319 | 0.00151 |
| 1359.355 | 0.00157 |
| 1358.39 | 0.00161 |
| 1357.426 | 0.00166 |
| 1356.462 | 0.00167 |
| 1355.498 | 0.00163 |
| 1354.533 | 0.0016 |
| 1353.569 | 0.00159 |
| 1352.605 | 0.00159 |
| 1351.641 | 0.0016 |
| 1350.676 | 0.00159 |
| 1349.712 | 0.00157 |
| 1348.748 | 0.00159 |
| 1347.783 | 0.00166 |
| 1346.819 | 0.00172 |
| 1345.855 | 0.00178 |
| 1344.891 | 0.0018 |
| 1343.926 | 0.00174 |
| 1342.962 | 0.00166 |
| 1341.998 | 0.00165 |
| 1341.034 | 0.00169 |
| 1340.069 | 0.00174 |
| 1339.105 | 0.00176 |
| 1338.141 | 0.0018 |
| 1337.177 | 0.00188 |
| 1336.212 | 0.00193 |
| 1335.248 | 0.00192 |
| 1334.284 | 0.00186 |
| 1333.319 | 0.00175 |
| 1332.355 | 0.0016 |
| 1331.391 | 0.00144 |
| 1330.427 | 0.00131 |
| 1329.462 | 0.00123 |
| 1328.498 | 0.00114 |
| 1327.534 | 0.00105 |
| 1326.57 | 0.001 |
| 1325.605 | 0.001 |
| 1324.641 | 0.00102 |
| 1323.677 | 0.00097 |
| 1322.713 | 0.0009 |
| 1321.748 | 0.00084 |
| 1320.784 | 0.00085 |
| 1319.82 | 0.00093 |
| 1318.855 | 0.00103 |
| 1317.891 | 0.00108 |
| 1316.927 | 0.00111 |
| 1315.963 | 0.00118 |
| 1314.998 | 0.00124 |
| 1314.034 | 0.00132 |
| 1313.07 | 0.00135 |
| 1312.106 | 0.00132 |
| 1311.141 | 0.00131 |
| 1310.177 | 0.00133 |
| 1309.213 | 0.00131 |
| 1308.248 | 0.00128 |
| 1307.284 | 0.00126 |
| 1306.32 | 0.00125 |
| 1305.356 | 0.00119 |
| 1304.391 | 0.00114 |
| 1303.427 | 0.00113 |
| 1302.463 | 0.00113 |
| 1301.499 | 0.00114 |
| 1300.534 | 0.00114 |
| 1299.57 | 0.00112 |
| 1298.606 | 0.00119 |
| 1297.642 | 0.00131 |
| 1296.677 | 0.00139 |
| 1295.713 | 0.0014 |
| 1294.749 | 0.00139 |
| 1293.784 | 0.00142 |
| 1292.82 | 0.00149 |
| 1291.856 | 0.00153 |
| 1290.892 | 0.00155 |
| 1289.927 | 0.00151 |
| 1288.963 | 0.00147 |
| 1287.999 | 0.00148 |
| 1287.035 | 0.00149 |
| 1286.07 | 0.00136 |
| 1285.106 | 0.00119 |
| 1284.142 | 0.00104 |
| 1283.178 | 0.00093 |
| 1282.213 | 0.00085 |
| 1281.249 | 0.00073 |
| 1280.285 | 0.00068 |
| 1279.32 | 0.00068 |
| 1278.356 | 0.00072 |
| 1277.392 | 0.00075 |
| 1276.428 | 0.0008 |
| 1275.463 | 0.00089 |
| 1274.499 | 0.00102 |
| 1273.535 | 0.00111 |
| 1272.571 | 0.00118 |
| 1271.606 | 0.00125 |
| 1270.642 | 0.0013 |
| 1269.678 | 0.00133 |
| 1268.714 | 0.00133 |
| 1267.749 | 0.00131 |
| 1266.785 | 0.00131 |
| 1265.821 | 0.00138 |
| 1264.856 | 0.00146 |
| 1263.892 | 0.00148 |
| 1262.928 | 0.00144 |
| 1261.964 | 0.00146 |
| 1260.999 | 0.00149 |
| 1260.035 | 0.00151 |
| 1259.071 | 0.00147 |
| 1258.107 | 0.00141 |
| 1257.142 | 0.00143 |
| 1256.178 | 0.00142 |
| 1255.214 | 0.00137 |
| 1254.249 | 0.00129 |
| 1253.285 | 0.00125 |
| 1252.321 | 0.00123 |
| 1251.357 | 0.00121 |
| 1250.392 | 0.00114 |
| 1249.428 | 0.00106 |
| 1248.464 | 0.00096 |
| 1247.5 | 0.00088 |
| 1246.535 | 0.00091 |
| 1245.571 | 0.00095 |
| 1244.607 | 0.001 |
| 1243.643 | 0.0011 |
| 1242.678 | 0.00121 |
| 1241.714 | 0.00131 |
| 1240.75 | 0.00138 |
| 1239.785 | 0.00149 |
| 1238.821 | 0.00161 |
| 1237.857 | 0.00167 |
| 1236.893 | 0.00168 |
| 1235.928 | 0.00171 |
| 1234.964 | 0.00177 |
| 1234 | 0.00182 |
| 1233.036 | 0.00184 |
| 1232.071 | 0.00187 |
| 1231.107 | 0.00184 |
| 1230.143 | 0.00182 |
| 1229.179 | 0.00181 |
| 1228.214 | 0.00173 |
| 1227.25 | 0.00165 |
| 1226.286 | 0.0016 |
| 1225.321 | 0.00154 |
| 1224.357 | 0.0015 |
| 1223.393 | 0.00143 |
| 1222.429 | 0.00128 |
| 1221.464 | 0.0011 |
| 1220.5 | 0.00097 |
| 1219.536 | 0.00093 |
| 1218.572 | 0.00089 |
| 1217.607 | 0.00084 |
| 1216.643 | 0.00079 |
| 1215.679 | 0.0007 |
| 1214.715 | 0.00058 |
| 1213.75 | 0.00053 |
| 1212.786 | 0.00056 |
| 1211.822 | 0.0007 |
| 1210.857 | 0.00084 |
| 1209.893 | 0.00095 |
| 1208.929 | 0.00104 |
| 1207.965 | 0.0011 |
| 1207 | 0.00116 |
| 1206.036 | 0.00122 |
| 1205.072 | 0.00126 |
| 1204.108 | 0.00136 |
| 1203.143 | 0.00149 |
| 1202.179 | 0.00164 |
| 1201.215 | 0.0018 |
| 1200.25 | 0.00189 |
| 1199.286 | 0.00194 |
| 1198.322 | 0.00195 |
| 1197.358 | 0.00198 |
| 1196.393 | 0.00203 |
| 1195.429 | 0.00202 |
| 1194.465 | 0.00203 |
| 1193.501 | 0.00207 |
| 1192.536 | 0.00209 |
| 1191.572 | 0.00212 |
| 1190.608 | 0.00218 |
| 1189.644 | 0.00224 |
| 1188.679 | 0.00229 |
| 1187.715 | 0.0023 |
| 1186.751 | 0.0023 |
| 1185.786 | 0.00228 |
| 1184.822 | 0.00218 |
| 1183.858 | 0.00206 |
| 1182.894 | 0.00197 |
| 1181.929 | 0.00193 |
| 1180.965 | 0.00193 |
| 1180.001 | 0.00193 |
| 1179.037 | 0.00192 |
| 1178.072 | 0.00191 |
| 1177.108 | 0.00181 |
| 1176.144 | 0.0017 |
| 1175.18 | 0.00162 |
| 1174.215 | 0.00158 |
| 1173.251 | 0.00154 |
| 1172.287 | 0.00153 |
| 1171.322 | 0.00157 |
| 1170.358 | 0.00156 |
| 1169.394 | 0.00152 |
| 1168.43 | 0.00152 |
| 1167.465 | 0.00153 |
| 1166.501 | 0.00155 |
| 1165.537 | 0.00157 |
| 1164.573 | 0.0016 |
| 1163.608 | 0.00161 |
| 1162.644 | 0.00158 |
| 1161.68 | 0.00153 |
| 1160.715 | 0.0015 |
| 1159.751 | 0.00149 |
| 1158.787 | 0.00155 |
| 1157.823 | 0.00159 |
| 1156.858 | 0.00156 |
| 1155.894 | 0.00154 |
| 1154.93 | 0.00154 |
| 1153.966 | 0.00152 |
| 1153.001 | 0.00153 |
| 1152.037 | 0.00153 |
| 1151.073 | 0.00145 |
| 1150.109 | 0.00137 |
| 1149.144 | 0.00132 |
| 1148.18 | 0.00124 |
| 1147.216 | 0.00118 |
| 1146.251 | 0.00118 |
| 1145.287 | 0.00122 |
| 1144.323 | 0.00124 |
| 1143.359 | 0.00125 |
| 1142.394 | 0.00127 |
| 1141.43 | 0.00132 |
| 1140.466 | 0.00143 |
| 1139.502 | 0.00157 |
| 1138.537 | 0.00166 |
| 1137.573 | 0.00175 |
| 1136.609 | 0.00181 |
| 1135.645 | 0.00185 |
| 1134.68 | 0.00188 |
| 1133.716 | 0.00186 |
| 1132.752 | 0.00185 |
| 1131.787 | 0.00188 |
| 1130.823 | 0.0019 |
| 1129.859 | 0.00189 |
| 1128.895 | 0.00186 |
| 1127.93 | 0.0018 |
| 1126.966 | 0.00172 |
| 1126.002 | 0.00164 |
| 1125.038 | 0.00155 |
| 1124.073 | 0.00152 |
| 1123.109 | 0.00151 |
| 1122.145 | 0.00146 |
| 1121.181 | 0.00137 |
| 1120.216 | 0.00129 |
| 1119.252 | 0.00125 |
| 1118.288 | 0.00126 |
| 1117.323 | 0.00131 |
| 1116.359 | 0.00133 |
| 1115.395 | 0.00131 |
| 1114.431 | 0.0013 |
| 1113.466 | 0.00129 |
| 1112.502 | 0.00136 |
| 1111.538 | 0.00144 |
| 1110.574 | 0.00146 |
| 1109.609 | 0.00145 |
| 1108.645 | 0.00145 |
| 1107.681 | 0.00146 |
| 1106.716 | 0.00152 |
| 1105.752 | 0.00159 |
| 1104.788 | 0.0016 |
| 1103.824 | 0.00159 |
| 1102.859 | 0.00161 |
| 1101.895 | 0.00166 |
| 1100.931 | 0.00169 |
| 1099.967 | 0.0017 |
| 1099.002 | 0.00171 |
| 1098.038 | 0.00173 |
| 1097.074 | 0.00174 |
| 1096.11 | 0.00176 |
| 1095.145 | 0.00178 |
| 1094.181 | 0.00174 |
| 1093.217 | 0.00171 |
| 1092.252 | 0.00173 |
| 1091.288 | 0.00177 |
| 1090.324 | 0.0018 |
| 1089.36 | 0.00182 |
| 1088.395 | 0.00177 |
| 1087.431 | 0.00171 |
| 1086.467 | 0.00167 |
| 1085.503 | 0.00164 |
| 1084.538 | 0.00165 |
| 1083.574 | 0.00164 |
| 1082.61 | 0.00156 |
| 1081.646 | 0.00143 |
| 1080.681 | 0.00131 |
| 1079.717 | 0.00128 |
| 1078.753 | 0.00127 |
| 1077.788 | 0.00124 |
| 1076.824 | 0.00121 |
| 1075.86 | 0.00116 |
| 1074.896 | 0.00113 |
| 1073.931 | 0.00106 |
| 1072.967 | 0.00104 |
| 1072.003 | 0.00107 |
| 1071.039 | 0.00111 |
| 1070.074 | 0.00119 |
| 1069.11 | 0.00123 |
| 1068.146 | 0.00124 |
| 1067.182 | 0.00126 |
| 1066.217 | 0.00128 |
| 1065.253 | 0.0013 |
| 1064.289 | 0.00136 |
| 1063.324 | 0.00146 |
| 1062.36 | 0.00157 |
| 1061.396 | 0.00169 |
| 1060.432 | 0.00175 |
| 1059.467 | 0.00174 |
| 1058.503 | 0.00176 |
| 1057.539 | 0.00187 |
| 1056.575 | 0.00201 |
| 1055.61 | 0.00212 |
| 1054.646 | 0.0022 |
| 1053.682 | 0.00222 |
| 1052.717 | 0.00221 |
| 1051.753 | 0.0022 |
| 1050.789 | 0.0022 |
| 1049.825 | 0.00218 |
| 1048.86 | 0.00214 |
| 1047.896 | 0.00208 |
| 1046.932 | 0.00197 |
| 1045.968 | 0.00179 |
| 1045.003 | 0.00164 |
| 1044.039 | 0.00152 |
| 1043.075 | 0.00138 |
| 1042.111 | 0.00128 |
| 1041.146 | 0.00123 |
| 1040.182 | 0.00117 |
| 1039.218 | 0.00109 |
| 1038.253 | 0.00102 |
| 1037.289 | 0.00093 |
| 1036.325 | 0.00087 |
| 1035.361 | 0.00085 |
| 1034.396 | 0.00089 |
| 1033.432 | 0.00086 |
| 1032.468 | 0.00084 |
| 1031.504 | 0.00086 |
| 1030.539 | 0.00086 |
| 1029.575 | 0.00085 |
| 1028.611 | 0.00087 |
| 1027.647 | 0.00092 |
| 1026.682 | 0.00098 |
| 1025.718 | 0.00109 |
| 1024.754 | 0.0012 |
| 1023.789 | 0.00126 |
| 1022.825 | 0.00129 |
| 1021.861 | 0.00138 |
| 1020.897 | 0.00144 |
| 1019.932 | 0.00145 |
| 1018.968 | 0.00144 |
| 1018.004 | 0.00136 |
| 1017.04 | 0.00126 |
| 1016.075 | 0.00119 |
| 1015.111 | 0.00118 |
| 1014.147 | 0.00117 |
| 1013.183 | 0.00115 |
| 1012.218 | 0.00113 |
| 1011.254 | 0.00113 |
| 1010.29 | 0.00109 |
| 1009.325 | 0.0011 |
| 1008.361 | 0.00111 |
| 1007.397 | 0.00111 |
| 1006.433 | 0.00114 |
| 1005.468 | 0.0012 |
| 1004.504 | 0.00131 |
| 1003.54 | 0.0014 |
| 1002.576 | 0.00144 |
| 1001.611 | 0.00149 |
| 1000.647 | 0.00153 |
| 999.6828 | 0.00156 |
| 998.7185 | 0.00157 |
| 997.7542 | 0.00153 |
| 996.7899 | 0.00142 |
| 995.8257 | 0.00129 |
| 994.8614 | 0.00117 |
| 993.8971 | 0.00111 |
| 992.9329 | 0.00112 |
| 991.9686 | 0.0011 |
| 991.0043 | 0.00107 |
| 990.0401 | 0.00103 |
| 989.0758 | 0.00096 |
| 988.1115 | 0.00086 |
| 987.1473 | 0.00075 |
| 986.183 | 0.0007 |
| 985.2187 | 0.00068 |
| 984.2545 | 0.00067 |
| 983.2902 | 0.0007 |
| 982.3259 | 0.0007 |
| 981.3617 | 0.00068 |
| 980.3974 | 0.00064 |
| 979.4331 | 0.00063 |
| 978.4689 | 0.00069 |
| 977.5046 | 0.00075 |
| 976.5403 | 0.00081 |
| 975.5761 | 0.00085 |
| 974.6118 | 0.00082 |
| 973.6475 | 0.00076 |
| 972.6832 | 0.00077 |
| 971.719 | 0.00081 |
| 970.7547 | 0.00085 |
| 969.7904 | 0.0009 |
| 968.8262 | 0.00093 |
| 967.8619 | 0.00102 |
| 966.8976 | 0.00111 |
| 965.9334 | 0.00113 |
| 964.9691 | 0.00113 |
| 964.0048 | 0.00118 |
| 963.0406 | 0.00123 |
| 962.0763 | 0.00127 |
| 961.112 | 0.00127 |
| 960.1478 | 0.00125 |
| 959.1835 | 0.00125 |
| 958.2192 | 0.00125 |
| 957.255 | 0.00121 |
| 956.2907 | 0.00117 |
| 955.3264 | 0.00116 |
| 954.3622 | 0.00118 |
| 953.3979 | 0.0012 |
| 952.4336 | 0.00115 |
| 951.4694 | 0.00107 |
| 950.5051 | 0.00106 |
| 949.5408 | 0.00106 |
| 948.5765 | 0.00104 |
| 947.6123 | 0.00108 |
| 946.648 | 0.00115 |
| 945.6837 | 0.00125 |
| 944.7195 | 0.00132 |
| 943.7552 | 0.00134 |
| 942.7909 | 0.00134 |
| 941.8267 | 0.00135 |
| 940.8624 | 0.00137 |
| 939.8981 | 0.00137 |
| 938.9339 | 0.00135 |
| 937.9696 | 0.00129 |
| 937.0053 | 0.00124 |
| 936.0411 | 0.00124 |
| 935.0768 | 0.00121 |
| 934.1125 | 0.00118 |
| 933.1483 | 0.00119 |
| 932.184 | 0.00125 |
| 931.2197 | 0.00136 |
| 930.2555 | 0.00146 |
| 929.2912 | 0.00149 |
| 928.3269 | 0.00149 |
| 927.3627 | 0.00152 |
| 926.3984 | 0.00154 |
| 925.4341 | 0.00158 |
| 924.4698 | 0.0016 |
| 923.5056 | 0.00159 |
| 922.5413 | 0.00157 |
| 921.577 | 0.00152 |
| 920.6128 | 0.00143 |
| 919.6485 | 0.00135 |
| 918.6842 | 0.00127 |
| 917.72 | 0.00121 |
| 916.7557 | 0.00115 |
| 915.7914 | 0.00109 |
| 914.8272 | 0.00104 |
| 913.8629 | 0.00101 |
| 912.8986 | 0.00101 |
| 911.9344 | 0.00104 |
| 910.9701 | 0.00111 |
| 910.0058 | 0.00115 |
| 909.0416 | 0.00123 |
| 908.0773 | 0.00128 |
| 907.113 | 0.00128 |
| 906.1488 | 0.00126 |
| 905.1845 | 0.00125 |
| 904.2202 | 0.00127 |
| 903.256 | 0.0013 |
| 902.2917 | 0.00129 |
| 901.3274 | 0.00127 |
| 900.3632 | 0.00124 |
| 899.3989 | 0.0012 |
| 898.4346 | 0.00112 |
| 897.4703 | 0.00103 |
| 896.5061 | 0.00098 |
| 895.5418 | 0.00092 |
| 894.5775 | 0.00083 |
| 893.6133 | 0.00076 |
| 892.649 | 0.00072 |
| 891.6847 | 0.00075 |
| 890.7205 | 0.00076 |
| 889.7562 | 0.0008 |
| 888.7919 | 0.00088 |
| 887.8277 | 0.00097 |
| 886.8634 | 0.00105 |
| 885.8991 | 0.00102 |
| 884.9349 | 0.00095 |
| 883.9706 | 0.00092 |
| 883.0063 | 0.00096 |
| 882.0421 | 0.00102 |
| 881.0778 | 0.00109 |
| 880.1135 | 0.00118 |
| 879.1493 | 0.00124 |
| 878.185 | 0.00127 |
| 877.2207 | 0.00129 |
| 876.2565 | 0.00125 |
| 875.2922 | 0.00115 |
| 874.3279 | 0.00111 |
| 873.3636 | 0.00109 |
| 872.3994 | 0.00105 |
| 871.4351 | 0.00103 |
| 870.4708 | 0.00106 |
| 869.5066 | 0.00115 |
| 868.5423 | 0.00127 |
| 867.578 | 0.00132 |
| 866.6138 | 0.00135 |
| 865.6495 | 0.00137 |
| 864.6852 | 0.00141 |
| 863.721 | 0.00143 |
| 862.7567 | 0.00139 |
| 861.7924 | 0.00135 |
| 860.8282 | 0.00133 |
| 859.8639 | 0.00132 |
| 858.8996 | 0.00135 |
| 857.9354 | 0.00141 |
| 856.9711 | 0.00145 |
| 856.0068 | 0.00141 |
| 855.0426 | 0.00137 |
| 854.0783 | 0.00133 |
| 853.114 | 0.00125 |
| 852.1498 | 0.00118 |
| 851.1855 | 0.00116 |
| 850.2212 | 0.00114 |
| 849.2569 | 0.00111 |
| 848.2927 | 0.0011 |
| 847.3284 | 0.0011 |
| 846.3641 | 0.00108 |
| 845.3999 | 0.00105 |
| 844.4356 | 0.00101 |
| 843.4713 | 0.00095 |
| 842.5071 | 0.00095 |
| 841.5428 | 0.00104 |
| 840.5785 | 0.0011 |
| 839.6143 | 0.00109 |
| 838.65 | 0.00106 |
| 837.6857 | 0.00106 |
| 836.7215 | 0.00114 |
| 835.7572 | 0.00126 |
| 834.7929 | 0.00133 |
| 833.8287 | 0.00134 |
| 832.8644 | 0.00132 |
| 831.9001 | 0.0013 |
| 830.9359 | 0.0013 |
| 829.9716 | 0.0013 |
| 829.0073 | 0.00133 |
| 828.0431 | 0.00138 |
| 827.0788 | 0.00141 |
| 826.1145 | 0.00146 |
| 825.1502 | 0.00146 |
| 824.186 | 0.00141 |
| 823.2217 | 0.00134 |
| 822.2574 | 0.00131 |
| 821.2932 | 0.00133 |
| 820.3289 | 0.00141 |
| 819.3646 | 0.0015 |
| 818.4004 | 0.00153 |
| 817.4361 | 0.0015 |
| 816.4718 | 0.00147 |
| 815.5076 | 0.00147 |
| 814.5433 | 0.00145 |
| 813.579 | 0.00141 |
| 812.6148 | 0.00136 |
| 811.6505 | 0.00131 |
| 810.6862 | 0.00131 |
| 809.722 | 0.00134 |
| 808.7577 | 0.00139 |
| 807.7934 | 0.00148 |
| 806.8292 | 0.00155 |
| 805.8649 | 0.00158 |
| 804.9006 | 0.00161 |
| 803.9364 | 0.00168 |
| 802.9721 | 0.00179 |
| 802.0078 | 0.00186 |
| 801.0436 | 0.00191 |
| 800.0793 | 0.00196 |
| 799.115 | 0.00202 |
| 798.1507 | 0.00211 |
| 797.1865 | 0.0022 |
| 796.2222 | 0.00223 |
| 795.2579 | 0.00221 |
| 794.2937 | 0.0022 |
| 793.3294 | 0.00217 |
| 792.3651 | 0.00213 |
| 791.4009 | 0.0021 |
| 790.4366 | 0.00209 |
| 789.4723 | 0.00208 |
| 788.5081 | 0.00208 |
| 787.5438 | 0.00203 |
| 786.5795 | 0.00197 |
| 785.6153 | 0.0019 |
| 784.651 | 0.00182 |
| 783.6867 | 0.00175 |
| 782.7225 | 0.00172 |
| 781.7582 | 0.00175 |
| 780.7939 | 0.00183 |
| 779.8297 | 0.0019 |
| 778.8654 | 0.00186 |
| 777.9011 | 0.00185 |
| 776.9369 | 0.00185 |
| 775.9726 | 0.0018 |
| 775.0083 | 0.00176 |
| 774.044 | 0.00175 |
| 773.0798 | 0.00172 |
| 772.1155 | 0.00169 |
| 771.1512 | 0.00168 |
| 770.187 | 0.00169 |
| 769.2227 | 0.00171 |
| 768.2584 | 0.00172 |
| 767.2942 | 0.00163 |
| 766.3299 | 0.0015 |
| 765.3656 | 0.00139 |
| 764.4014 | 0.00129 |
| 763.4371 | 0.00125 |
| 762.4728 | 0.00119 |
| 761.5086 | 0.00113 |
| 760.5443 | 0.00115 |
| 759.58 | 0.00112 |
| 758.6158 | 0.00106 |
| 757.6515 | 0.00106 |
| 756.6872 | 0.00106 |
| 755.723 | 0.00099 |
| 754.7587 | 0.00095 |
| 753.7944 | 0.00086 |
| 752.8302 | 0.00074 |
| 751.8659 | 0.0007 |
| 750.9016 | 0.0007 |
| 749.9373 | 0.00067 |
| 748.9731 | 0.0007 |
| 748.0088 | 0.00077 |
| 747.0445 | 0.00082 |
| 746.0803 | 0.00085 |
| 745.116 | 0.00087 |
| 744.1517 | 0.00087 |
| 743.1875 | 0.00095 |
| 742.2232 | 0.00106 |
| 741.2589 | 0.00113 |
| 740.2947 | 0.00116 |
| 739.3304 | 0.00118 |
| 738.3661 | 0.00124 |
| 737.4019 | 0.00125 |
| 736.4376 | 0.0012 |
| 735.4733 | 0.00122 |
| 734.5091 | 0.00128 |
| 733.5448 | 0.00126 |
| 732.5805 | 0.00115 |
| 731.6163 | 0.00103 |
| 730.652 | 0.00096 |
| 729.6877 | 0.00092 |
| 728.7235 | 0.00089 |
| 727.7592 | 0.00087 |
| 726.7949 | 0.00088 |
| 725.8306 | 0.00089 |
| 724.8664 | 0.0009 |
| 723.9021 | 0.00087 |
| 722.9378 | 0.00086 |
| 721.9736 | 0.00091 |
| 721.0093 | 0.00104 |
| 720.045 | 0.00121 |
| 719.0808 | 0.00132 |
| 718.1165 | 0.00134 |
| 717.1522 | 0.00136 |
| 716.188 | 0.00139 |
| 715.2237 | 0.00141 |
| 714.2594 | 0.00135 |
| 713.2952 | 0.00131 |
| 712.3309 | 0.00123 |
| 711.3666 | 0.00111 |
| 710.4024 | 0.00099 |
| 709.4381 | 0.00092 |
| 708.4738 | 0.00089 |
| 707.5096 | 0.00087 |
| 706.5453 | 0.00085 |
| 705.581 | 0.00084 |
| 704.6168 | 0.00087 |
| 703.6525 | 0.0009 |
| 702.6882 | 0.00085 |
| 701.724 | 0.00079 |
| 700.7597 | 0.00075 |
| 699.7954 | 0.00078 |
| 698.8311 | 0.00094 |
| 697.8669 | 0.00112 |
| 696.9026 | 0.00122 |
| 695.9383 | 0.00128 |
| 694.9741 | 0.00132 |
| 694.0098 | 0.00135 |
| 693.0455 | 0.00135 |
| 692.0813 | 0.00129 |
| 691.117 | 0.0012 |
| 690.1527 | 0.00114 |
| 689.1885 | 0.0011 |
| 688.2242 | 0.00114 |
| 687.2599 | 0.00117 |
| 686.2957 | 0.00117 |
| 685.3314 | 0.00116 |
| 684.3671 | 0.00117 |
| 683.4029 | 0.00119 |
| 682.4386 | 0.00118 |
| 681.4743 | 0.00116 |
| 680.5101 | 0.00111 |
| 679.5458 | 0.00106 |
| 678.5815 | 0.00104 |
| 677.6173 | 0.00107 |
| 676.653 | 0.0012 |
| 675.6887 | 0.00139 |
| 674.7244 | 0.00152 |
| 673.7602 | 0.00159 |
| 672.7959 | 0.00167 |
| 671.8316 | 0.00174 |
| 670.8674 | 0.00179 |
| 669.9031 | 0.00176 |
| 668.9388 | 0.00172 |
| 667.9746 | 0.00172 |
| 667.0103 | 0.00173 |
| 666.046 | 0.00172 |
| 665.0818 | 0.00167 |
| 664.1175 | 0.00157 |
| 663.1532 | 0.00148 |
| 662.189 | 0.00141 |
| 661.2247 | 0.00129 |
| 660.2604 | 0.00114 |
| 659.2962 | 0.00097 |
| 658.3319 | 0.00081 |
| 657.3676 | 0.00069 |
| 656.4034 | 0.0006 |
| 655.4391 | 0.00051 |
| 654.4748 | 0.00047 |
| 653.5106 | 0.00051 |
| 652.5463 | 0.00059 |
| 651.582 | 0.00066 |
| 650.6177 | 0.0007 |
| 649.6535 | 0.00074 |
| 648.6892 | 0.00077 |
| 647.7249 | 0.00076 |
| 646.7607 | 0.00074 |
| 645.7964 | 0.00079 |
| 644.8321 | 0.00089 |
| 643.8679 | 0.00098 |
| 642.9036 | 0.00103 |
| 641.9393 | 0.00111 |
| 640.9751 | 0.00119 |
| 640.0108 | 0.00125 |
| 639.0465 | 0.00127 |
| 638.0823 | 0.00127 |
| 637.118 | 0.00128 |
| 636.1537 | 0.00131 |
| 635.1895 | 0.00133 |
| 634.2252 | 0.00133 |
| 633.2609 | 0.00132 |
| 632.2967 | 0.0013 |
| 631.3324 | 0.00132 |
| 630.3681 | 0.00134 |
| 629.4039 | 0.00133 |
| 628.4396 | 0.00127 |
| 627.4753 | 0.00118 |
| 626.511 | 0.00106 |
| 625.5468 | 0.00093 |
| 624.5825 | 0.0008 |
| 623.6182 | 0.00074 |
| 622.654 | 0.00075 |
| 621.6897 | 0.00076 |
| 620.7254 | 0.00074 |
| 619.7612 | 0.00072 |
| 618.7969 | 0.00076 |
| 617.8326 | 0.00084 |
| 616.8684 | 0.00091 |
| 615.9041 | 0.00099 |
| 614.9398 | 0.00106 |
| 613.9756 | 0.00113 |
| 613.0113 | 0.00121 |
| 612.047 | 0.00132 |
| 611.0828 | 0.00139 |
| 610.1185 | 0.00145 |
| 609.1542 | 0.00151 |
| 608.19 | 0.00158 |
| 607.2257 | 0.00158 |
| 606.2614 | 0.00153 |
| 605.2972 | 0.00142 |
| 604.3329 | 0.00128 |
| 603.3686 | 0.00115 |
| 602.4044 | 0.00105 |
| 601.4401 | 0.00101 |
| 600.4758 | 0.00101 |
| 599.5115 | 0.001 |
| 598.5473 | 0.00098 |
| 597.583 | 0.00097 |
| 596.6187 | 0.00096 |
| 595.6545 | 0.00095 |
| 594.6902 | 0.00094 |
| 593.7259 | 0.00094 |
| 592.7617 | 0.00095 |
| 591.7974 | 0.00096 |
| 590.8331 | 0.00103 |
| 589.8689 | 0.00114 |
| 588.9046 | 0.00128 |
| 587.9403 | 0.00136 |
| 586.9761 | 0.00138 |
| 586.0118 | 0.00138 |
| 585.0475 | 0.00134 |
| 584.0833 | 0.00129 |
| 583.119 | 0.00126 |
| 582.1547 | 0.00125 |
| 581.1905 | 0.00128 |
| 580.2262 | 0.00135 |
| 579.2619 | 0.00141 |
| 578.2977 | 0.00145 |
| 577.3334 | 0.00149 |
| 576.3691 | 0.0015 |
| 575.4048 | 0.00151 |
| 574.4406 | 0.00151 |
| 573.4763 | 0.00147 |
| 572.512 | 0.0014 |
| 571.5478 | 0.00132 |
| 570.5835 | 0.00126 |
| 569.6192 | 0.00125 |
| 568.655 | 0.00125 |
| 567.6907 | 0.00127 |
| 566.7264 | 0.00127 |
| 565.7622 | 0.00126 |
| 564.7979 | 0.0012 |
| 563.8336 | 0.00112 |
| 562.8694 | 0.00107 |
| 561.9051 | 0.00106 |
| 560.9408 | 0.00107 |
| 559.9766 | 0.00107 |
| 559.0123 | 0.00106 |
| 558.048 | 0.00106 |
| 557.0838 | 0.00108 |
| 556.1195 | 0.00114 |
| 555.1552 | 0.00125 |
| 554.191 | 0.00139 |
| 553.2267 | 0.00151 |
| 552.2624 | 0.0016 |
| 551.2981 | 0.00165 |
| 550.3339 | 0.00166 |
| 549.3696 | 0.00163 |
| 548.4053 | 0.00161 |
| 547.4411 | 0.0016 |
| 546.4768 | 0.00166 |
| 545.5125 | 0.00175 |
| 544.5483 | 0.0018 |
| 543.584 | 0.0018 |
| 542.6197 | 0.00175 |
| 541.6555 | 0.00165 |
| 540.6912 | 0.00153 |
| 539.7269 | 0.00144 |
| 538.7627 | 0.00138 |
| 537.7984 | 0.00137 |
| 536.8341 | 0.00133 |
| 535.8699 | 0.00126 |
| 534.9056 | 0.00122 |
| 533.9413 | 0.00125 |
| 532.9771 | 0.00132 |
| 532.0128 | 0.0014 |
| 531.0485 | 0.00147 |
| 530.0843 | 0.00153 |
| 529.12 | 0.00159 |
| 528.1557 | 0.00161 |
| 527.1914 | 0.00161 |
| 526.2272 | 0.00166 |
| 525.2629 | 0.00173 |
| 524.2986 | 0.00184 |
| 523.3344 | 0.00197 |
| 522.3701 | 0.00208 |
| 521.4058 | 0.00216 |
| 520.4416 | 0.0022 |
| 519.4773 | 0.00219 |
| 518.513 | 0.00217 |
| 517.5488 | 0.0022 |
| 516.5845 | 0.00226 |
| 515.6202 | 0.00228 |
| 514.656 | 0.00225 |
| 513.6917 | 0.0022 |
| 512.7274 | 0.00223 |
| 511.7632 | 0.00233 |
| 510.7989 | 0.00245 |
| 509.8346 | 0.00257 |
| 508.8704 | 0.00269 |
| 507.9061 | 0.00284 |
| 506.9418 | 0.003 |
| 505.9776 | 0.00312 |
| 505.0133 | 0.0032 |
| 504.049 | 0.00329 |
| 503.0848 | 0.00342 |
| 502.1205 | 0.00355 |
| 501.1562 | 0.00365 |
| 500.1919 | 0.00377 |
| 499.2277 | 0.00393 |
| 498.2634 | 0.00409 |
| 497.2991 | 0.00418 |
| 496.3349 | 0.00423 |
| 495.3706 | 0.00425 |
| 494.4063 | 0.00422 |
| 493.4421 | 0.00413 |
| 492.4778 | 0.00406 |
| 491.5135 | 0.00408 |
| 490.5493 | 0.0042 |
| 489.585 | 0.00436 |
| 488.6207 | 0.0045 |
| 487.6565 | 0.0046 |
| 486.6922 | 0.00465 |
| 485.7279 | 0.00467 |
| 484.7637 | 0.0047 |
| 483.7994 | 0.00471 |
| 482.8351 | 0.00473 |
| 481.8709 | 0.00476 |
| 480.9066 | 0.00482 |
| 479.9423 | 0.00492 |
| 478.9781 | 0.00501 |
| 478.0138 | 0.00511 |
| 477.0495 | 0.0052 |
| 476.0852 | 0.00526 |
| 475.121 | 0.00525 |
| 474.1567 | 0.00518 |
| 473.1924 | 0.00505 |
| 472.2282 | 0.00483 |
| 471.2639 | 0.00459 |
| 470.2996 | 0.00439 |
| 469.3354 | 0.00429 |
| 468.3711 | 0.00421 |
| 467.4068 | 0.0041 |
| 466.4426 | 0.00397 |
| 465.4783 | 0.00381 |
| 464.514 | 0.00362 |
| 463.5498 | 0.00347 |
| 462.5855 | 0.00347 |
| 461.6212 | 0.00362 |
| 460.657 | 0.00383 |
| 459.6927 | 0.00406 |
| 458.7284 | 0.0043 |
| 457.7642 | 0.00455 |
| 456.7999 | 0.00478 |
| 455.8356 | 0.00499 |
| 454.8714 | 0.00523 |
| 453.9071 | 0.00548 |
| 452.9428 | 0.00574 |
| 451.9785 | 0.00595 |
| 451.0143 | 0.0061 |
| 450.05 | 0.00617 |
| 449.0857 | 0.00617 |
| 448.1215 | 0.00615 |
| 447.1572 | 0.00613 |
| 446.1929 | 0.00606 |
| 445.2287 | 0.00593 |
| 444.2644 | 0.00578 |
| 443.3001 | 0.00567 |
| 442.3359 | 0.00556 |
| 441.3716 | 0.00545 |
| 440.4073 | 0.00539 |
| 439.4431 | 0.00538 |
| 438.4788 | 0.0054 |
| 437.5145 | 0.00542 |
| 436.5503 | 0.00544 |
| 435.586 | 0.00547 |
| 434.6217 | 0.00556 |
| 433.6575 | 0.00572 |
| 432.6932 | 0.00592 |
| 431.7289 | 0.0061 |
| 430.7647 | 0.00621 |
| 429.8004 | 0.00626 |
| 428.8361 | 0.00625 |
| 427.8718 | 0.00623 |
| 426.9076 | 0.00619 |
| 425.9433 | 0.00616 |
| 424.979 | 0.00615 |
| 424.0148 | 0.00615 |
| 423.0505 | 0.00614 |
| 422.0862 | 0.00611 |
| 421.122 | 0.00607 |
| 420.1577 | 0.006 |
| 419.1934 | 0.00589 |
| 418.2292 | 0.0058 |
| 417.2649 | 0.00577 |
| 416.3006 | 0.00579 |
| 415.3364 | 0.00579 |
| 414.3721 | 0.00575 |
| 413.4078 | 0.00565 |
| 412.4436 | 0.00558 |
| 411.4793 | 0.00553 |
| 410.515 | 0.00547 |
| 409.5508 | 0.00536 |
| 408.5865 | 0.00524 |
| 407.6222 | 0.00517 |
| 406.658 | 0.00514 |
| 405.6937 | 0.00514 |
| 404.7294 | 0.00511 |
| 403.7652 | 0.0051 |
| 402.8009 | 0.00515 |
| 401.8366 | 0.00522 |
| 400.8723 | 0.0053 |
| 399.9081 | 0.00537 |
| 398.9438 | 0.00546 |
| 397.9795 | 0.0056 |
| 397.0153 | 0.0058 |
| 396.051 | 0.00605 |
| 395.0867 | 0.00626 |
| 394.1225 | 0.00635 |
| 393.1582 | 0.00633 |
| 392.1939 | 0.00627 |
| 391.2297 | 0.00623 |
| 390.2654 | 0.00619 |
| 389.3011 | 0.00615 |
| 388.3369 | 0.00611 |
| 387.3726 | 0.00605 |
| 386.4083 | 0.00597 |
| 385.4441 | 0.00591 |
| 384.4798 | 0.00586 |
| 383.5155 | 0.0058 |
| 382.5513 | 0.00568 |
| 381.587 | 0.00553 |
| 380.6227 | 0.0054 |
| 379.6585 | 0.00531 |
| 378.6942 | 0.00523 |
| 377.7299 | 0.00512 |
| 376.7656 | 0.00502 |
| 375.8014 | 0.00495 |
| 374.8371 | 0.00493 |
| 373.8728 | 0.00494 |
| 372.9086 | 0.00497 |
| 371.9443 | 0.00498 |
| 370.98 | 0.00497 |
| 370.0158 | 0.00501 |
| 369.0515 | 0.0051 |
| 368.0872 | 0.00519 |
| 367.123 | 0.00524 |
| 366.1587 | 0.00525 |
| 365.1944 | 0.00526 |
| 364.2302 | 0.00526 |
| 363.2659 | 0.0053 |
| 362.3016 | 0.00539 |
| 361.3374 | 0.0055 |
| 360.3731 | 0.00553 |
| 359.4088 | 0.00543 |
| 358.4446 | 0.00527 |
| 357.4803 | 0.00513 |
| 356.516 | 0.00503 |
| 355.5518 | 0.0049 |
| 354.5875 | 0.00477 |
| 353.6232 | 0.0047 |
| 352.6589 | 0.00465 |
| 351.6947 | 0.0046 |
| 350.7304 | 0.00452 |
| 349.7661 | 0.00444 |
| 348.8019 | 0.00436 |
| 347.8376 | 0.0043 |
| 346.8733 | 0.00428 |
| 345.9091 | 0.00427 |
| 344.9448 | 0.00422 |
| 343.9805 | 0.00413 |
| 343.0163 | 0.00406 |
| 342.052 | 0.00404 |
| 341.0877 | 0.00408 |
| 340.1235 | 0.00413 |
| 339.1592 | 0.00415 |
| 338.1949 | 0.00413 |
| 337.2307 | 0.00407 |
| 336.2664 | 0.00399 |
| 335.3021 | 0.00394 |
| 334.3379 | 0.00397 |
| 333.3736 | 0.00405 |
| 332.4093 | 0.00416 |
| 331.4451 | 0.00431 |
| 330.4808 | 0.00445 |
| 329.5165 | 0.00451 |
| 328.5522 | 0.00455 |
| 327.588 | 0.00459 |
| 326.6237 | 0.00465 |
| 325.6594 | 0.00473 |
| 324.6952 | 0.00484 |
| 323.7309 | 0.00494 |
| 322.7666 | 0.00497 |
| 321.8024 | 0.00491 |
| 320.8381 | 0.00483 |
| 319.8738 | 0.00476 |
| 318.9096 | 0.00468 |
| 317.9453 | 0.00453 |
| 316.981 | 0.00435 |
| 316.0168 | 0.00423 |
| 315.0525 | 0.00417 |
| 314.0882 | 0.00411 |
| 313.124 | 0.004 |
| 312.1597 | 0.00393 |
| 311.1954 | 0.00393 |
| 310.2312 | 0.00399 |
| 309.2669 | 0.00404 |
| 308.3026 | 0.00403 |
| 307.3384 | 0.00401 |
| 306.3741 | 0.00399 |
| 305.4098 | 0.00402 |
| 304.4456 | 0.00405 |
| 303.4813 | 0.00405 |
| 302.517 | 0.00403 |
| 301.5527 | 0.00403 |
| 300.5885 | 0.00403 |
| 299.6242 | 0.00398 |
| 298.6599 | 0.00391 |
| 297.6957 | 0.00383 |
| 296.7314 | 0.00373 |
| 295.7671 | 0.0036 |
| 294.8029 | 0.00349 |
| 293.8386 | 0.00348 |
| 292.8743 | 0.00354 |
| 291.9101 | 0.0036 |
| 290.9458 | 0.00365 |
| 289.9815 | 0.0037 |
| 289.0173 | 0.00377 |
| 288.053 | 0.00384 |
| 287.0887 | 0.0039 |
| 286.1245 | 0.0039 |
| 285.1602 | 0.00384 |
| 284.1959 | 0.00376 |
| 283.2317 | 0.00369 |
| 282.2674 | 0.00359 |
| 281.3031 | 0.00344 |
| 280.3389 | 0.00327 |
| 279.3746 | 0.00312 |
| 278.4103 | 0.00301 |
| 277.446 | 0.00295 |
| 276.4818 | 0.00288 |
| 275.5175 | 0.00281 |
| 274.5532 | 0.00274 |
| 273.589 | 0.00267 |
| 272.6247 | 0.00259 |
| 271.6604 | 0.0025 |
| 270.6962 | 0.00243 |
| 269.7319 | 0.00239 |
| 268.7676 | 0.00237 |
| 267.8034 | 0.00241 |
| 266.8391 | 0.00245 |
| 265.8748 | 0.00248 |
| 264.9106 | 0.0025 |
| 263.9463 | 0.0025 |
| 262.982 | 0.00248 |
| 262.0178 | 0.00245 |
| 261.0535 | 0.00243 |
| 260.0892 | 0.00242 |
| 259.125 | 0.00242 |
| 258.1607 | 0.00244 |
| 257.1964 | 0.00251 |
| 256.2322 | 0.0026 |
| 255.2679 | 0.00271 |
| 254.3036 | 0.00279 |
| 253.3393 | 0.00282 |
| 252.3751 | 0.00284 |
| 251.4108 | 0.00289 |
| 250.4465 | 0.00296 |
| 249.4823 | 0.00297 |
| 248.518 | 0.00294 |
| 247.5537 | 0.00295 |
| 246.5895 | 0.00301 |
| 245.6252 | 0.00307 |
| 244.6609 | 0.0031 |
| 243.6967 | 0.00309 |
| 242.7324 | 0.00306 |
| 241.7681 | 0.00299 |
| 240.8039 | 0.00291 |
| 239.8396 | 0.00281 |
| 238.8753 | 0.00272 |
| 237.9111 | 0.00263 |
| 236.9468 | 0.00258 |
| 235.9825 | 0.00254 |
| 235.0183 | 0.00248 |
| 234.054 | 0.0024 |
| 233.0897 | 0.0023 |
| 232.1255 | 0.0022 |
| 231.1612 | 0.00215 |
| 230.1969 | 0.00216 |
| 229.2326 | 0.00223 |
| 228.2684 | 0.00231 |
| 227.3041 | 0.00237 |
| 226.3398 | 0.0024 |
| 225.3756 | 0.00241 |
| 224.4113 | 0.00244 |
| 223.447 | 0.00248 |
| 222.4828 | 0.00251 |
| 221.5185 | 0.00256 |
| 220.5542 | 0.0026 |
| 219.59 | 0.00259 |
| 218.6257 | 0.00253 |
| 217.6614 | 0.00243 |
| 216.6972 | 0.00232 |
| 215.7329 | 0.00224 |
| 214.7686 | 0.00218 |
| 213.8044 | 0.00213 |
| 212.8401 | 0.00203 |
| 211.8758 | 0.00188 |
| 210.9116 | 0.00171 |
| 209.9473 | 0.0016 |
| 208.983 | 0.00156 |
| 208.0188 | 0.00154 |
| 207.0545 | 0.0015 |
| 206.0902 | 0.00143 |
| 205.126 | 0.00136 |
| 204.1617 | 0.00137 |
| 203.1974 | 0.00141 |
| 202.2331 | 0.00143 |
| 201.2689 | 0.00143 |
| 200.3046 | 0.00148 |
| 199.3403 | 0.00159 |
| 198.3761 | 0.00167 |
| 197.4118 | 0.0017 |
| 196.4475 | 0.00167 |
| 195.4833 | 0.00165 |
| 194.519 | 0.00168 |
| 193.5547 | 0.00171 |
| 192.5905 | 0.00176 |
| 191.6262 | 0.00183 |
| 190.6619 | 0.00187 |
| 189.6977 | 0.00185 |
| 188.7334 | 0.00178 |
| 187.7691 | 0.00174 |
| 186.8049 | 0.0017 |
| 185.8406 | 0.00162 |
| 184.8763 | 0.00154 |
| 183.9121 | 0.00151 |
| 182.9478 | 0.00149 |
| 181.9835 | 0.00149 |
| 181.0193 | 0.0015 |
| 180.055 | 0.00144 |
| 179.0907 | 0.00131 |
| 178.1264 | 0.00119 |
| 177.1622 | 0.00116 |
| 176.1979 | 0.00122 |
| 175.2336 | 0.00131 |
| 174.2694 | 0.00141 |
| 173.3051 | 0.00152 |
| 172.3408 | 0.00162 |
| 171.3766 | 0.00168 |
| 170.4123 | 0.00171 |
| 169.448 | 0.00177 |
| 168.4838 | 0.00186 |
| 167.5195 | 0.00197 |
| 166.5552 | 0.00208 |
| 165.591 | 0.00216 |
| 164.6267 | 0.00218 |
| 163.6624 | 0.00217 |
| 162.6982 | 0.00215 |
| 161.7339 | 0.00212 |
| 160.7696 | 0.00201 |
| 159.8054 | 0.00183 |
| 158.8411 | 0.00166 |
| 157.8768 | 0.00151 |
| 156.9126 | 0.00136 |
| 155.9483 | 0.00123 |
| 154.984 | 0.00122 |
| 154.0197 | 0.00132 |
| 153.0555 | 0.00144 |
| 152.0912 | 0.00151 |
| 151.1269 | 0.00156 |
| 150.1627 | 0.00155 |
| 149.1984 | 0.00147 |
| 148.2341 | 0.00139 |
| 147.2699 | 0.00136 |
| 146.3056 | 0.00138 |
| 145.3413 | 0.00142 |
| 144.3771 | 0.00146 |
| 143.4128 | 0.00149 |
| 142.4485 | 0.00145 |
| 141.4843 | 0.00139 |
| 140.52 | 0.00134 |
| 139.5557 | 0.00123 |
| 138.5915 | 0.00105 |
| 137.6272 | 0.0009 |
| 136.6629 | 0.00084 |
| 135.6987 | 0.00088 |
| 134.7344 | 0.00092 |
| 133.7701 | 0.00092 |
| 132.8059 | 0.00093 |
| 131.8416 | 0.00098 |
| 130.8773 | 0.00105 |
| 129.913 | 0.00112 |
| 128.9488 | 0.00121 |
| 127.9845 | 0.00128 |
| 127.0202 | 0.0013 |
| 126.056 | 0.00133 |
| 125.0917 | 0.0014 |
| 124.1274 | 0.00146 |
| 123.1632 | 0.00151 |
| 122.1989 | 0.00156 |
| 121.2346 | 0.00164 |
| 120.2704 | 0.00176 |
| 119.3061 | 0.00185 |
| 118.3418 | 0.00192 |
| 117.3776 | 0.00196 |
| 116.4133 | 0.00193 |
| 115.449 | 0.00193 |
| 114.4848 | 0.00201 |
| 113.5205 | 0.00214 |
| 112.5562 | 0.00222 |
| 111.592 | 0.00223 |
| 110.6277 | 0.0022 |
| 109.6634 | 0.00212 |
| 108.6992 | 0.00205 |
| 107.7349 | 0.00202 |
| 106.7706 | 0.00201 |
| 105.8064 | 0.00198 |
| 104.8421 | 0.00195 |
| 103.8778 | 0.00192 |
| 102.9135 | 0.00187 |
| 101.9493 | 0.00179 |
| 100.985 | 0.00171 |
| 100.0207 | 0.00165 |
| 99.05647 | 0.0016 |
| 98.0922 | 0.00158 |
| 97.12793 | 0.00155 |
| 96.16367 | 0.00148 |
| 95.1994 | 0.00138 |
| 94.23513 | 0.00133 |
| 93.27086 | 0.00136 |
| 92.30659 | 0.00141 |
| 91.34233 | 0.0015 |
| 90.37806 | 0.00154 |
| 89.41379 | 0.00154 |
| 88.44952 | 0.00153 |
| 87.48525 | 0.00148 |
| 86.52099 | 0.00141 |
| 85.55672 | 0.0014 |
| 84.59245 | 0.00147 |
| 83.62818 | 0.00156 |
| 82.66391 | 0.00162 |
| 81.69965 | 0.00163 |
| 80.73538 | 0.00159 |
| 79.77111 | 0.00147 |
| 78.80684 | 0.00135 |
| 77.84257 | 0.00128 |
| 76.87831 | 0.00123 |
| 75.91404 | 0.00123 |
| 74.94977 | 0.0013 |
| 73.9855 | 0.00144 |
| 73.02124 | 0.0016 |
| 72.05697 | 0.00179 |
| 71.0927 | 0.00199 |
| 70.12843 | 0.00214 |
| 69.16416 | 0.00219 |
| 68.1999 | 0.0022 |
| 67.23563 | 0.00217 |
| 66.27136 | 0.00212 |
| 65.30709 | 0.00204 |
| 64.34282 | 0.00196 |
| 63.37856 | 0.00186 |
| 62.41429 | 0.00186 |
| 61.45002 | 0.00186 |
| 60.48575 | 0.00186 |
| 59.52148 | 0.00186 |
| 58.55722 | 0.00186 |
| 57.59295 | 0.00186 |
| 56.62868 | 0.00186 |
| 55.66441 | 0.00186 |
| 54.70014 | 0.00186 |
| 53.73588 | 0.00186 |
| 52.77161 | 0.00186 |
| 51.80734 | 0.00186 |
